# Correction of global physiology in resting-state functional near-infrared spectroscopy

**DOI:** 10.1117/1.NPh.9.3.035003

**Published:** 2022-08-18

**Authors:** Pradyumna Lanka, Heather Bortfeld, Theodore J. Huppert

**Affiliations:** a University of California, Merced, Department of Psychological Sciences, Merced, California, United States; b University of California, Merced, Department of Cognitive and Information Sciences, Merced, California, United States; c University of Pittsburgh, Department of Electrical and Computer Engineering, Pittsburgh, Pennsylvania, United States

**Keywords:** functional near-infrared spectroscopy, functional connectivity, Granger causality, resting-state analysis

## Abstract

**Significance:**

Resting-state functional connectivity (RSFC) analyses of functional near-infrared spectroscopy (fNIRS) data reveal cortical connections and networks across the brain. Motion artifacts and systemic physiology in evoked fNIRS signals present unique analytical challenges, and methods that control for systemic physiological noise have been explored. Whether these same methods require modification when applied to resting-state fNIRS (RS-fNIRS) data remains unclear.

**Aim:**

We systematically examined the sensitivity and specificity of several RSFC analysis pipelines to identify the best methods for correcting global systemic physiological signals in RS-fNIRS data.

**Approach:**

Using numerically simulated RS-fNIRS data, we compared the rates of true and false positives for several connectivity analysis pipelines. Their performance was scored using receiver operating characteristic analysis. Pipelines included partial correlation and multivariate Granger causality, with and without short-separation measurements, and a modified multivariate causality model that included a non-traditional zeroth-lag cross term. We also examined the effects of pre-whitening and robust statistical estimators on performance.

**Results:**

Consistent with previous work on bivariate correlation models, our results demonstrate that robust statistics and pre-whitening are effective methods to correct for motion artifacts and autocorrelation in the fNIRS time series. Moreover, we found that pre-filtering using principal components extracted from short-separation fNIRS channels as part of a partial correlation model was most effective in reducing spurious correlations due to shared systemic physiology when the two signals of interest fluctuated synchronously. However, when there was a temporal lag between the signals, a multivariate Granger causality test incorporating the short-separation channels was better. Since it is unknown if such a lag exists in experimental data, we propose a modified version of Granger causality that includes the non-traditional zeroth-lag term as a compromising solution.

**Conclusions:**

A combination of pre-whitening, robust statistical methods, and partial correlation in the processing pipeline to reduce autocorrelation, motion artifacts, and global physiology are suggested for obtaining statistically valid connectivity metrics with RS-fNIRS. Further studies should validate the effectiveness of these methods using human data.

## 1 Introduction

Spontaneous or resting-state functional connectivity (RSFC) has been an important tool for characterizing the network architecture of the human brain. Biswal et al.^1^ first noted that synchronized spontaneous fluctuations in the functional magnetic resonance imaging (fMRI) blood-oxygen-level-dependent (BOLD) signals were organized into distinct networks that were conserved across subjects. More than 25 years later, these results have been replicated in hundreds of research studies and expanded to nearly every aspect of human brain function, development, and disease.^2^^–^^8^ Resting-state networks have also been observed using other neuroimaging modalities, including electroencephalography and magnetoencephalography,^9^^,^^10^ as well as functional near-infrared spectroscopy (fNIRS).^11^^,^^12^ A major advantage of the resting-state paradigm is that there is no task compliance necessary, which is especially useful in research with infant, aging, diseased, and other non-compliant populations.

Not surprisingly, several analytical methods have been developed to quantify resting-state connectivity. For both fMRI and fNIRS, these methods often focus on the zeroth-lag relationship between brain regions. Zeroth-lag relationships are ones in which signal fluctuations occur simultaneously (within the measurement sampling time) in both signals. Perhaps, the most common method for estimating functional connectivity is to calculate the Pearson correlation coefficient between the time series from two brain regions. There are several ways to characterize RSFC in the time-domain, including (i) seed-based connectivity, (ii) pairwise connectivity, and (iii) independent component analysis (ICA). In seed-based connectivity, a seed region is selected, and its connectivity with all other brain regions is assessed.^13^ In pairwise connectivity, the correlation between all possible pairs of channels is calculated.^14^ ICA decomposes the covariance of fNIRS channel data into several statistically independent spatiotemporal components, with some components representing functional networks in the brain either for each subject individually or for the entire dataset concatenated across subjects.^15^^,^^16^

Granger causality is an example of an “effective connectivity” method, which relies on the lagged relationship between fNIRS channels in the time-domain. Granger causality analysis (GCA) is based on the principle of Granger causality, which states that for two signals, x and y, if the past values of signal x contain information that helps predict y above and beyond the information contained in past values of y alone, then we can say x Granger-causes y.^17^^,^^18^ The term “causal” refers to the mathematical predictability that the history of the time course of x has to the current value of y, rather than the more linguistic context of cause and effect. GCA is statistically tested using a likelihood ratio test comparing the goodness-of-fit of a linear model containing both x and y terms to a restricted model containing only y terms. In the case of a multivariate model, this is a likelihood ratio test of a model containing {x, y, and other confounds Z} to one containing only {y and other confounds Z}. GCA is especially suited for modeling time-lagged relationships between brain regions. It benefits from computational simplicity without the need to prespecify the direction of influence, unlike the other effective connectivity methods, including structural equation modeling and dynamic causal modeling, which are more confirmatory than exploratory. Although several studies have looked at Granger causality in task-based fNIRS paradigms,^19^^–^^21^ there is a paucity of studies that examine GCA in resting-state paradigms.

Another method commonly used to model functional connectivity in fNIRS is the wavelet transform coherence (WTC), which models the relationship between the channels in the time-frequency domain. WTC relies on the continuous wavelet transform (CWT). CWT is the convolution of the scaled and shifted versions of a mother wavelet, and transforms the time series data into the time-frequency domain. Often the Morlet wavelet, a Gaussian scaled sine wave, is chosen as the mother wavelet. WTC is estimated as a cross-correlation between the time series in the time-frequency domain.^22^^–^^24^

### 1.1 Functional Near-Infrared Spectroscopy

The fNIRS is an optical imaging method that uses light in the near-infrared wavelength range (650 to 950 nm) to estimate temporal changes in the concentrations of oxyhemoglobin (HbO) and deoxyhemoglobin (HbR).^25^ In this range of red to near-infrared light, the differential optical absorption of HbO and HbR allows for estimation of hemodynamic signals in the brain through a set of optical sources and detectors placed non-invasively on the scalp of participants (e.g., Refs. 26 and 27). Light from these optical sources passes through the skull and scatters through the tissue and can reach the outer few millimeters of the cerebral cortex allowing detection of underlying brain signals. Thus, fNIRS measures the hemodynamic changes in the brain reflecting changes in blood flow, volume, and blood oxygenation, and therefore, is an indirect measure of neural activity like the fMRI BOLD signal. FNIRS has some advantages compared to fMRI including lower cost, a greater resiliency to head motion (provided the optical sensors remain secured to the scalp), and higher data sampling rates (although still measuring slow hemodynamic signals). FNIRS is also portable and is commercially available in participant-wearable systems, which allows subject movement, application to a wider range of populations that would have contraindications in other modalities, and the ability to perform more flexible or ecologically valid tasks.

However, the disadvantages of fNIRS are its lower spatial resolution, limited depth penetration into the brain, and increased sensitivity to superficial layers of the head relative to the brain itself. The latter issue is particularly confounding for fNIRS measurements, since systemic physiology in the scalp, including cardiac, respiratory, and blood pressure fluctuations, often result in spatially global noise, which is often larger in amplitude than the underlying brain signals of interest.^28^^,^^29^ Thus, in the case of RSFC using fNIRS, the separation of the overlying spatial networks due to superficial physiological signals and true underlying neural-related brain networks is challenging.

The first study to demonstrate the feasibility of fNIRS in capturing the very-low-frequency and low-frequency oscillations that contribute to RSFC was by Obrig et al.^30^ After that, several early studies used to investigate the feasibility of RSFC found reliable and expected visual, sensorimotor, and language networks using fNIRS and diffuse optical tomography (DOT).^13^^,^^31^^–^^33^ Since then, researchers have used resting-state fNIRS (RS-fNIRS) and DOT to study infant development,^14^ gender differences in the prefrontal cortex,^34^ preterm birth,^35^ language networks,^36^ autism spectrum disorder,^37^^,^^38^ affective disorders,^39^ and aging.^40^ Studies examining the test-retest reliability of RSFC have found good to excellent intraclass correlations across both individual and group level RSFC maps across multiple sessions ranging from an hour to a week.^41^^,^^42^ More importantly, RSFC with fNIRS (RS-fNIRS) has also been validated with RS-fMRI.^43^^,^^44^ Although extremely popular with fMRI, just a few papers have applied graph-theoretic approaches to RS-fNIRS.^41^^,^^45^

### 1.2 Statistical Properties of RS-fNIRS Signal

RS-fNIRS has some unique challenges compared to RS-fMRI given the signal and noise properties of RS-fNIRS. Due to the way the fNIRS signal is collected, it is sensitive to non-neural extracerebral signal changes in the scalp. RS-fNIRS signal is composed of three components: (i) low-frequency neural oscillations (signal of interest), (ii) intracerebral physiological noise originating in the brain, and (iii) extracerebral physiological noise originating in the scalp and non-brain tissues. All the signals are non-evoked, given the nature of the resting-state paradigm. Thus, fNIRS signals are contaminated by both extracerebral signal changes as well as non-neural cerebral signal changes due to systemic physiology attributed to cardiac (around 1 to 1.2 Hz), respiratory (0.3 to 0.6 Hz), and blood pressure/Mayer wave (0.1 Hz) fluctuations.^29^^,^^46^

Physiological noise has two effects on the RS-fNIRS signal: (i) inducing temporal autocorrelation and (ii) increasing spatial covariance between channels across the brain. Both these issues and how they affect the statistical assumptions of the connectivity models used to identify the interactions between time series will be discussed in detail below. The proposed ways to correct for them will be discussed in the subsequent sections.

#### 1.2.1 Temporal autocorrelation

Previous work has demonstrated that the high sampling rate and the systemic physiology (heart rate, respiration, and blood pressure), combined with the underlying hemodynamic response, can together lead to temporal dependencies, and hence autocorrelation in RS-fNIRS time series.^47^ Colored noise reduces the effective degrees of freedom. Autocorrelation may be present for up to several seconds, thus, lasting multiple time points depending on the sampling rate.^47^^,^^48^ In this case, the assumption of independence of cases is no longer valid and using linear regression/correlation to model the relationship between autocorrelated time series could lead to increased false positives.^49^ The reason for the increased false positives can be attributed to the underestimation of standard errors of the null distribution of the regression coefficients, as well as a possible bias in the estimates (if the autocorrelation structures are different across the time series) due to the temporal autocorrelations in the time series,^50^ neither of which are considered when drawing statistical inferences.

An effective way to remove the autocorrelation in the time series to obtain more statistically valid estimates of relationships is a procedure called pre-whitening. Pre-whitening works by removing the serial correlations in the time series. Autoregressive (AR) models can be used for pre-whitening the data and removing the serial correlations. Specifically, for fNIRS, using pre-whitening entails using a p’th order AR model to model the current timepoint as a function of the previous timepoints. The residual term in the AR model after removing the temporal dependencies is termed the innovation term, and is now used to calculate the Pearson correlation coefficient between channels rather than using the autocorrelated channel data.^47^ In a study comparing the use of standard correlation without pre-whitening and AR correlation with pre-whitening on simulated data, Santosa et al.^47^ reported a false discovery rate (FDR) as high as 50% at 1-Hz sampling rate and increasing to 70% for a sampling rate of 10 Hz when the standard correlation is used to model the functional connectivity between fNIRS time series. With the use of AR-correlation with pre-whitening incorporated, however, the uncontrolled FDR reduced to the expected 5% at 1-Hz sampling rate and a higher 30% FDR at 10-Hz sampling rate. Similar findings were also reported for experimental fNIRS data, thus demonstrating a reduced type-I error rate when pre-whitening is incorporated into the preprocessing pipeline for estimation of functional connectivity.^47^ Despite correcting for the temporal autocorrelations in fNIRS, Santosa et al.^47^ did not account for the increased spatial covariance due to systemic physiology, which could also lead to increased FDR.

#### 1.2.2 Global systemic physiology

Since systemic physiology is thought to be relatively homogenous across the brain, the signal changes caused by the systemic fluctuations that are shared across all the channels can increase the spatial covariance in the channels.^52^ This can artificially inflate the strength of the relationship between the time series due to a third term (physiological noise). Figure 1 shows that not correcting for the increased spatial covariance in the fNIRS data could lead to increased type-I errors and reduced specificity, as far more connectivity paths are present than expected based on the “ground truth.”

**Fig. 1:**
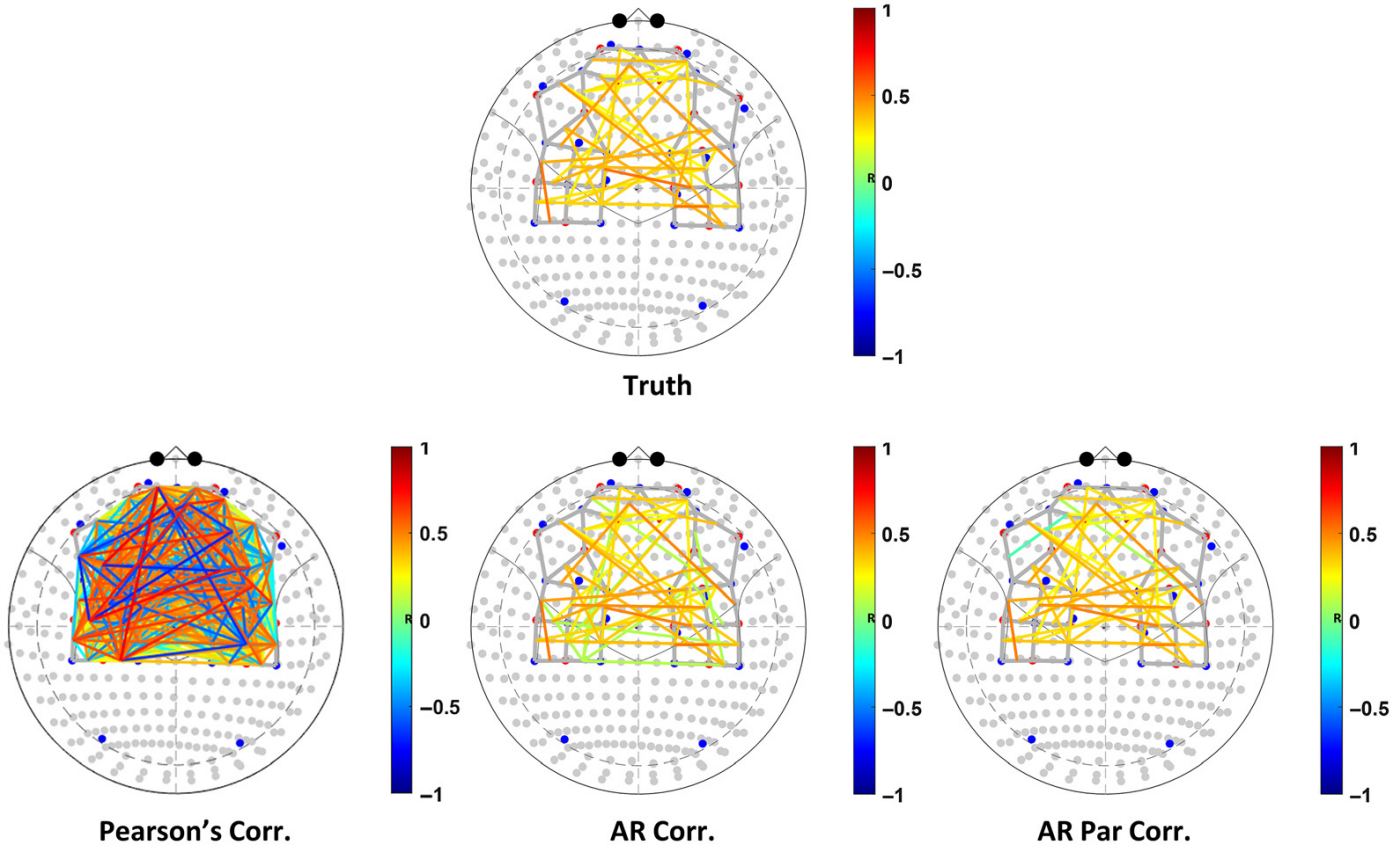
Connectivity maps show the effectiveness of (i) Pearson correlation coefficient, (ii) AR correlation, and (iii) AR partial correlation, to recover true connectivity, in simulated fNIRS data with added physiological noise mimicking AR smoothing and shared covariance. The figure demonstrates the success of AR partial correlation to recover most of the true connectivity patterns in simulated data with fewer false positives.

#### 1.2.3 Proposed solutions to correct for the effects of physiological noise

Several solutions have been proposed to deal with reducing the influence of physiological noise on the violations of assumptions of connectivity due to them. Most methods that have been proposed to remove the effects of physiological data do not account for the autocorrelation in the fNIRS time series, but instead aim to improve the signal-to-noise ratio by removing the influence of physiological noise and non-neural signals.

Generally, the most common method to reduce the interference of physiological noise in fNIRS data is bandpass filtering. Brain-derived signals, exhibit a power spectral density in accordance with the inverse-power law with greater power at lower frequencies and much less power at higher frequencies.^53^ It must be noted that with fNIRS, we are measuring the hemodynamic/vascular changes associated with neural activity rather than the neural activity itself, so the hemodynamic response function (HRF) acts as a temporal low-pass filter. Given the inverse-power law and the low-pass filtering properties of HRF, there is little high-frequency information present in the RS-fNIRS signal. Finally, the fNIRS signal is also corrupted by high-frequency noise structures such as cardiac and respiratory noise, thus a good signal-to-noise ratio of neural activity compared to noise is often restricted to low-frequency bands in fNIRS. The fNIRS signal also generally has very-low-frequency drift noise, which can be another source of autocorrelation. The very low-frequency drift noise can be modeled and removed by linear, polynomial, or discrete cosine transform basis functions, or by filtering the data with an appropriate high-pass filter. Thus, using an appropriate bandpass filter could remove physiological noise, while still preserving the low-frequency neural oscillations.^28^ It must be noted that low-pass filtering/bandpass filtering often increases the autocorrelation in the time series, whilst reducing the degrees of freedom. Consequently, low-pass/bandpass filtering also increases the false positive rate.^54^

An alternative approach to removing spatial noise is to use spatial principal component [principal components analysis (PCA)] filtering.^28^^,^^55^ PCA depends on the idea that shared spatial covariance between the signals is due to shared physiology, and the variance is much larger in magnitude compared to the neural signal. So, the first few components obtained from PCA can be used to extract the spatially correlated global signal. Regressing out the PCA components from the fNIRS data provides a way to reduce the shared global signal due to systemic physiology, while still preserving neural-related components.

Still another way to reduce the presence of the physiological signals is to use external recording devices to measure respiration, heart rate, and blood pressure, and remove their effects from the RS-fNIRS signal.^27^^,^^56^^,^^57^ However, this method is often expensive as it requires using additional instruments to record the physiological signals. Moreover, reducing physiological noise may not entirely remove the autocorrelation in the time series. More recently, the use of short-separation channels has become a popular approach to estimate local systemic physiology. Because the depth of penetration of light into the tissue is a function of the spacing between the fNIRS light source and detector positions, typically for measuring the brain, a spacing of about 3 to 4 cm is used, which provides penetration to a few millimeters into the cortex. When short-separation (∼5 to 10 mm) spacings are used, the light only reaches into the scalp and can be used as a regressor of superficial physiology.^58^ The use of these short-separation fNIRS measurements has been shown to be an effective way to remove the global effects of systemic physiology for evoked (task-based) functional signals.^51^

Previously, several approaches using these short-separation channel measurements been proposed, including as pre-filtering operators or as regressors within linear regression models.^51^^,^^58^^–^^62^ However, shorter distance channels can introduce engineering challenges when another detector has to be placed so close to the source, and it can also cause saturation issues.^63^ Placing the detector too far from the source can introduce false negatives in the data because the short-separation channel can capture neural-related variance. Studies have identified the optimum distance for a short-separation channel for adults is 8.4 and 2.15 mm for infants using Monte Carlo simulations.^63^ Meanwhile, hybrid approaches that use short-separation channels together with PCA appear to be more effective in reducing systemic physiology.^64^^,^^65^

One final method for reducing the impact of global systemic physiology on fNIRS-based RSFC data is to use partial correlation instead of Pearson correlation coefficient. Partial correlation is better at removing the shared covariance between the signals and is more sensitive to the relationship between deep brain signals that reflect true neural connectivity.^52^ Connectivity values obtained from the partial correlation between channels are lower than those obtained from Pearson correlation due to the removal of extracerebral contributions to the connectivity. However, using partial correlation by controlling for other channels can also often remove neural variance as well, thus, increasing the rate of false negatives.

#### 1.2.4 Head motion artifacts

In addition to physiological noise, motion artifacts are another source of error in fNIRS data. Motion artifacts occur when the head movements of the participant cause the optodes to slide or momentarily lose contact with the scalp, leading to spikes in signal intensity or changes in baseline signal intensity.^66^ Motion artifacts are common in datasets from certain populations, such as infants and young children, as well as in experimental paradigms that require movement. Usually, the signal changes induced by head motion are much larger than the signal changes due to neural activity.^67^ Motion artifacts can affect only a few probes or most of the probes, thus increasing or reducing the shared covariance between the channel time series.^47^

The simplest way to address the effect of motion artifacts is to remove the motion corrupted timepoints from analysis, although, it has been argued that correcting for motion rather than simply removing the motion corrupted timepoints is a better strategy.^66^ For RS-fNIRS, completely removing motion corrupted timepoints from analysis may reduce the stability of connectivity metrics due to the reduced number of timepoints. Methods to correct for motion artifacts include spline interpolation,^68^ wavelet filtering,^69^ PCA,^28^^,^^55^ discrete Kalman filtering,^70^ and correlation-based signal improvement.^71^ Although these methods are discussed in detail in Brigadoi et al., in the context of task-based analyses,^66^ they are also applicable to RS-fNIRS data. For task-based fNIRS analysis, wavelet filtering was identified as the best-performing method relative to the other options.^66^ Since then, several other methods and improvements to existing methods have been proposed with varying degrees of success in correcting for multiple manifestations of motion artifacts in the fNIRS data. Some of those methods include kurtosis-based wavelet filtering,^72^ temporal derivative distribution and repair ^73^ and global variance of temporal derivatives-based motion censoring.^74^

Alternatively, motion artifacts in the fNIRS data can be corrected within the context of a generalized linear model using robust statistics. Head motion present in the resting-state data violates the assumptions of non-normality, as the motion-contaminated timepoints could appear as outliers in the distribution of the innovation terms.^47^^,^^54^^,^^67^ Using pre-weighting by downweighting timepoints considered outliers can reduce the impact of the motion contaminated timepoints on calculated connectivity metrics such as correlation.^47^ Santosa et al.^47^ proposed a general linear model (GLM) that was robust to the violations of assumptions using methods such as pre-whitening and pre-weighting such that the inferences drawn about the parameters were still statistically valid, unlike traditional methods that do not explicitly account for the violation of assumptions.

### 1.3 Aim

In this paper, we examine the performance of several approaches to correcting systemic physiology and global signal artifacts for RS-fNIRS as a tool to track functional connectivity between different brain regions. To this end, we quantified the sensitivity/specificity and false positive rates of various numerical methods, including several approaches that use short-separation fNIRS measurements as either pre-filtering/partial correlation methods, as well as within multivariate correlation and causal models. This work is a multi-channel/multivariate expansion of our previous examination of bivariate correlation analysis for fNIRS.^47^ Santosa et al.^47^ demonstrated that the slow hemodynamic signals measured by fNIRS result in spurious correlations, very high false positive rates, and uncorrected type-I error in the standard Pearson correlation model unless corrected for noise autocorrelation and other non-spherical statistical errors. However, their work did not address the issue of spatially correlated noise due to superficial systemic physiology, the topic of the current research.

## 2 Methods

To characterize the sensitivity and specificity of each of the proposed analysis methods, we relied on numerically simulated data with a known ground truth. We explored the model performance over several classes of simulations ranging from simple normally distributed random noise (where all the statistical assumptions of our model are valid) to “physiological” signals with temporally autocorrelated noise to global spatial physiological noise. Finally, we included the presence of motion artifacts. Each step introduced additional violations to the statistical assumptions of the model, requiring modifications for model generalization. Each data simulation represented a more challenging (and experimentally plausible) statistical problem, which we examined using receiver operating characteristics (ROC) analysis by simulating true and null correlations in the data. All of the methods were implemented in the NIRS AnalyzIR toolbox^75^ using MATLAB (R2019b) (The MathWorks, Inc., Natick, Massachusetts).

### 2.1 Data Simulation

FNIRS data were simulated for a probe consisting of 16 long-distance channels (27 mm) and nine short-separation channels (7.1 mm) using a multivariate normal distribution with a specified spatial covariance matrix. Data were simulated assuming near-infrared wavelengths of 690 and 830 nm. This known spatial covariance generates the underlying “true” correlations in the data, upon which the various algorithms are assessed. These true correlations were generated with either a zero lag (no time shift) and/or a temporal lag between channels (mathematically causal model). The target true correlations were simulated in channel-space for only the long-distance channels. To add additional noise to the model, a semi-infinite homogenous slab model was used to compute the optical forward model (sensitivity of each channel to the underlying brain volume). Noise was added to the voxels in the first 2.5-mm modeling a superficial skin layer. For some simulations, superficial noise was temporal and/or spatially smoothed as detailed as follows. The optical forward model was then used to project these voxels to channel space and added to the base truth correlation signals.

For each simulation, we generated 300 s of resting-state data at a 4-Hz sample rate. To generate the ROC curves, a total of 200 data sets were generated for each test. In the ROC analysis, of all possible connections between any two channels in the probe, true connections were simulated for around 10% for the possible connectivity paths, ensuring that global systemic noise was the dominant spatial noise feature. So only 10% of the non-diagonal elements in the covariance matrix used for generating the simulated data were non-zeros. Any significant connections between the remaining 90% of the paths are false positives due to temporal autocorrelation, global systemic physiology, or motion artifacts. To generate true and false positive estimates, an equal number of true and null connections were randomly taken from the total adjacency matrix.

#### 2.1.1 Random noise simulations

For the initial simulations, the normally distributed, white noise model described already was used to generate the ROC curves. No additional superficial noise was added to the data. Thus, this set of simulations lacked any realistic physiological noise and consisted of both temporally and spatially uncorrelated noise. However, this set of simulations is consistent with the statistical assumptions of the standard Pearson correlation model, which assumes normally distributed, independent, random samples. Results for random noise simulation are not shown.

#### 2.1.2 Physiological temporal noise simulations

In the second set of simulations, serially correlated noise structures were introduced to mimic the temporal structure of physiological noise. In the voxels of the “skin” layer of the model, a random noise model was convolved with an auto-regressive noise structure of model order 10 to generate physiologically colored noise. These signals were then projected through the optical forward model to channel-space and added to the base true correlation signals. For this set of simulations, only temporally structured noise was added as there was no spatial structure to the noise across channels. The ratio of the simulated neural components to the superficial simulated physiological noise was 1:1 in the simulated data. The signal-to-noise ratio of the neural signal to the random white noise in these simulations was 100:1 (40 dB). The white noise models the shot noise and the signal-to-noise ratio is in the range previously reported.^76^^,^^77^

#### 2.1.3 Physiological temporal and spatial noise simulations

In the third set of simulations, an additional spatial noise structure was introduced in addition to temporal autocorrelation. In this set of simulations, the skin layer voxels were spatially smoothed using a Gaussian spatial kernel with a full-width half-max of 150 mm. These voxels were projected into channel space to generate a “global” systemic noise model. Since we used the optical forward model, the projection of this noise to the long-distance and short-separation NIRS channels was consistent with the skin origins of these signals. As with earlier simulation, the ratio of the simulated neural signal to the superficial simulated physiological noise was 1:1 in the simulated data and the ratio of neural signal to the white noise was 100:1.

#### 2.1.4 Motion artifact simulations

Two types of motion artifact were added to the time series to mimic the motion artifacts encountered in fNIRS time series: shift artifacts and spike artifacts. These artifacts were added to the base simulations described already (random noise, temporal noise, or spatial and temporal noise). The simulated motion artifacts that were added were similar to the motion effects observed in child imaging studies with moderate levels of head motion.^78^

*Shift artifacts*. The rate of shift artifacts added to the data was around 0.5 per minute or 1 shift artifact for every 480 timepoints if the data are assumed to be sampled at 4 Hz. The shift artifact was modeled as a scalar shift in amplitude added to the time series sampled from a normal distribution with a zero mean and a standard deviation of five times the original data.

*Spike artifacts*. Similarly, the rate of spike artifacts added to the data was around 2 per minute or 1 spike artifact for every 120 timepoints of the data for data sampled at 4 Hz. The spike artifact was modeled with a Laplacian distribution function with the peak amplitude sampled from a normal distribution with a mean zero and a standard deviation of five times the standard deviation of the original data. The scale parameter was randomly sampled from a uniform distribution of 0.05 to 5. This ensures that the spike artifact occurs at the same time across multiple channels, but the amplitude of the spike artifact varies across the channels.

### 2.2 Experimental fNIRS Data

#### 2.2.1 Acquisition

We also wanted to test the effectiveness and applicability of our functional connectivity methods to experimental resting-state data. NIRS data were recorded using a commercial NIRScout-2 (NIRx Medizintechnik GmbH, Berlin, Germany) continuous-wave fNIRS system with short-separation measurements. A total of 50 channels (42 channels for long-distance and 8 channels for short separation measurements) were distributed across the bilateral frontal and sensorimotor cortices (see Fig. 2). Long-distance channels comprised 16 source optodes (orange circles) and 13 detector optodes (blue rectangles) placed on the scalp, as shown in Fig. 2. A detector optode split into eight detectors (green diamonds) was used for short-separation channels in eight locations across the scalp. The solid line represents long-distance and the green dotted line represents the short-separation channels. The distances between source and detector were 23 to 50- and 7.5-mm for long-distance and short-separation channels, respectively, with the light blue solid line in Fig. 2 representing the channels. Data for two wavelengths (760 and 850 nm) were recorded at a sampling rate of 7.8125 Hz. After positioning the headcap, signal quality was optimized using the NIRx Aurora software. The ambient light was blocked using an opaque, black shower cap placed on the participant’s head during acquisition.

**Fig. 2:**
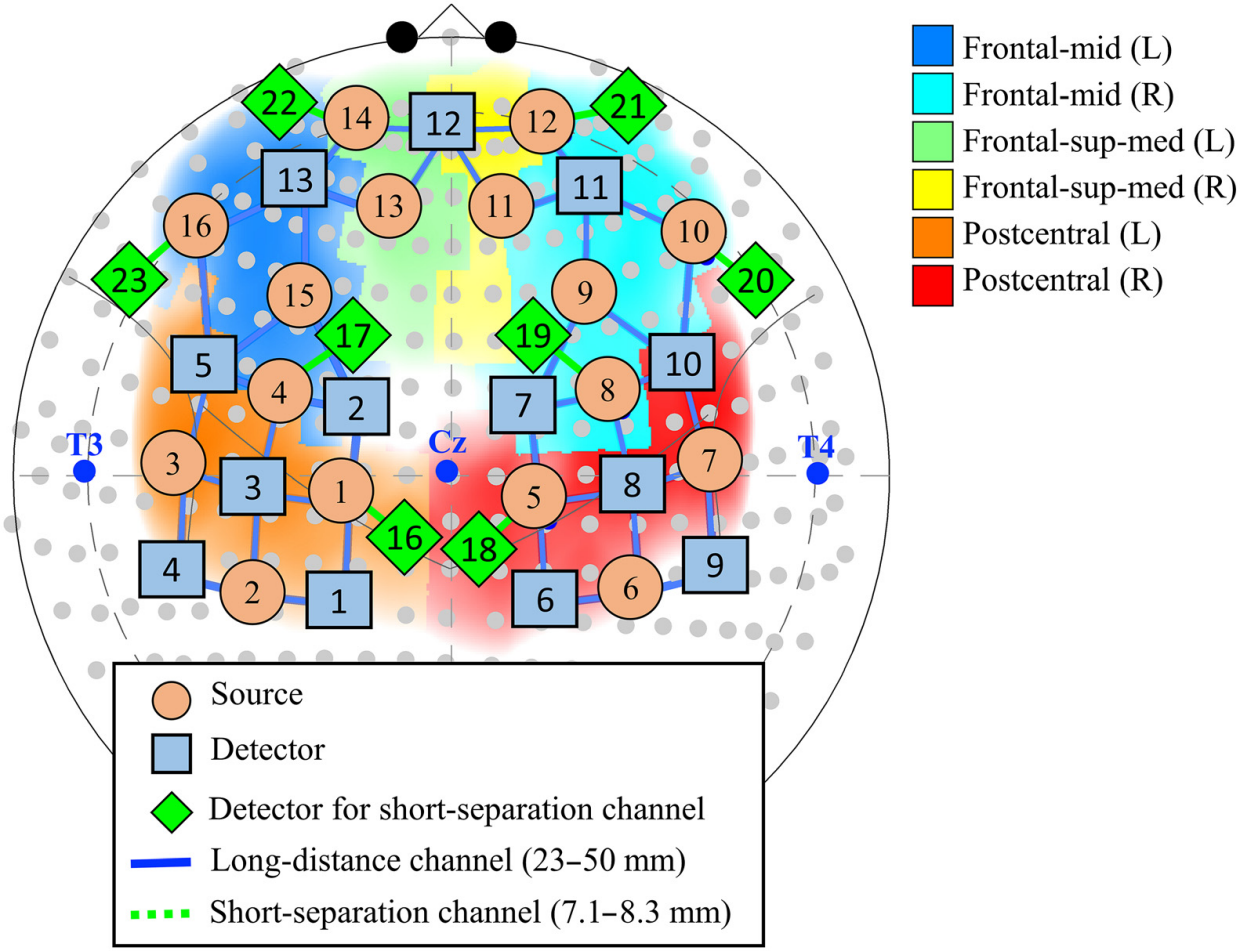
The schematic shows the placement of probes on the bilateral frontal and sensorimotor cortices superimposed on a 10–10 coordinate system for the experimental fNIRS data. About 16 sources and 13 detectors optodes formed a total of 42 long-distance channels. Additionally, a detector optode was split into eight short-separation detectors to give eight short-separation channels. The data acquired from these probes in 24 subjects were used to compare the effectiveness of zero-lag correlation methods.

#### 2.2.2 Participants & task

About 24 healthy subjects participated in the experiment (9 males and 15 females; mean age 27.4 years, s.d. 8.7 years; 23 right-handed). The subjects were informed about the experimentation and written consent was obtained. The study was approved by the University of Pittsburgh Institutional Review Board. Each subject performed one session of the resting-state scan along with other task scans. The subjects were instructed to minimize body motion and remain relaxed in the sitting position for 5 min without employing any mental effort for the duration of the resting-state scan.

### 2.3 Data Pre-Processing

We converted simulated fNIRS signal intensities to changes in optical density, and then converted optical density units to HbO and HbR concentrations using the modified Beer–Lambert law (MBLL) with a differential path length factor of 6 and a partial volume correction of 60 for both wavelengths. Data were preprocessed in a similar manner compared to the simulated data except for downsampling the experimental fNIRS time series. After the raw signal intensity was converted to HbO and HbR concentrations using MBLL, the experimental resting-state fNIRS data were downsampled to 4 Hz from 7.8125 Hz to match the sampling rate of the simulated data. For the remainder of the article, the analyses focus on just changes in the HbO concentrations and the findings should be applicable for HbR as well.

Figure 3 shows an example fNIRS time series from the simulated data for three conditions, (i) just temporal autocorrelation, (ii) with both temporal autocorrelation and global systemic physiology, and (iii) with both temporal autocorrelation, global systemic physiology, and motion artifacts. Specifically, left panels of Fig. 3 show the time traces of two channels for the three conditions after converting the raw simulated fNIRS signals to HbO signal changes using MBLL. The right panels of Fig. 3 show the correlation structure of the channels. As expected, there is increased covariance in the channels with the introduction of global systemic physiology. The covariance further increases drastically with correlation values between channels close to 1, with the introduction of the motion artifacts in the simulated data.

**Fig. 3:**
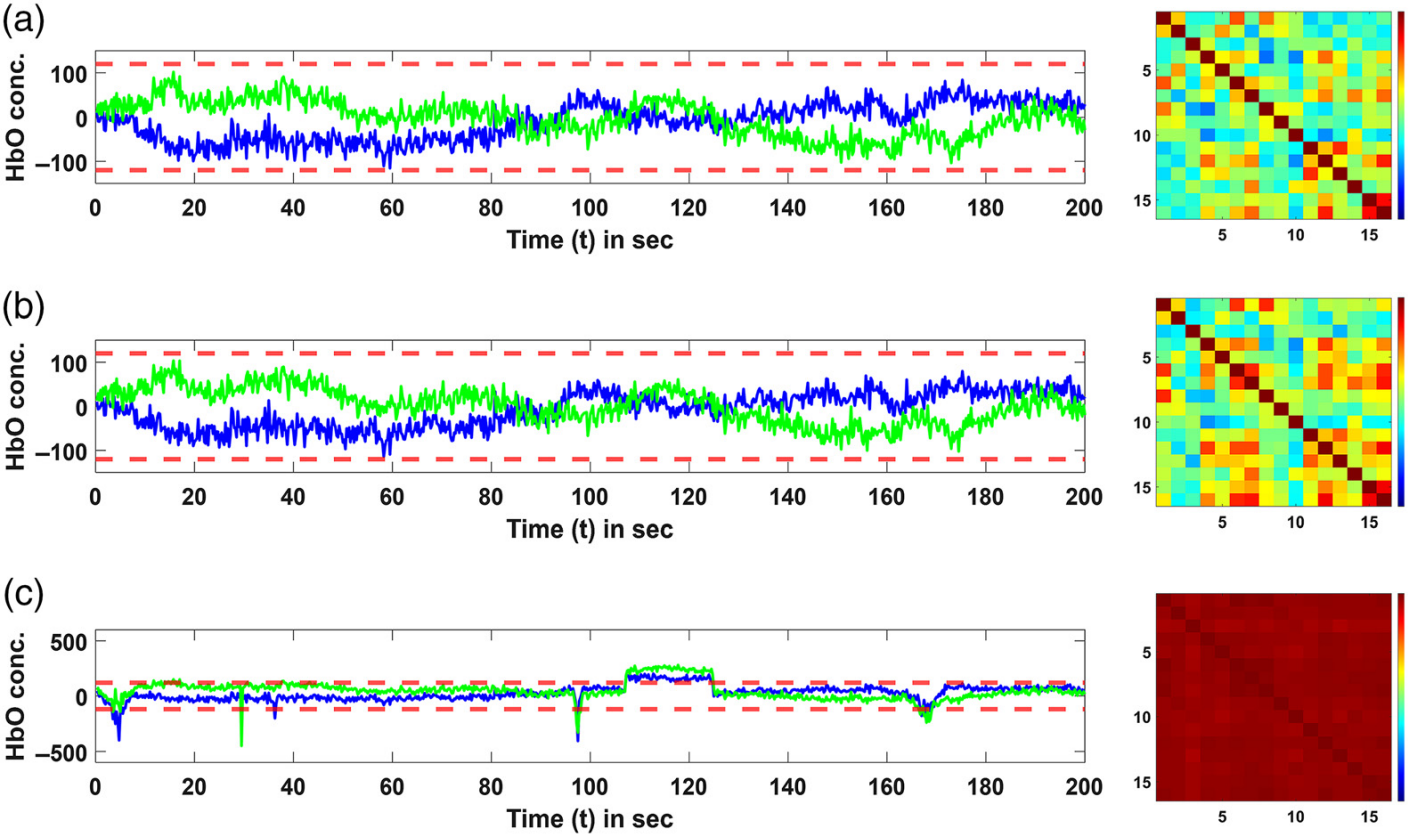
Plots showing the time traces of HbO changes for two representative channels in the simulated data as well as the normalized covariance matrices for the channels. Rows show simulated data with just (a) temporal autocorrelation; (b) with both temporal autocorrelation and global systemic physiology; and (c) with temporal autocorrelation, global systemic physiology, and motion artifacts. The panels on the left show the time series for two representative channels for 200 s and the panels on the right show the normalized covariance matrix of the HbO signal changes for all the 16 long-distance channels in the data.

#### 2.3.1 Pre-whitening

Pre-whitening often uses AR models to remove the autocorrelation in the time series and whitens the frequency content of the signal. Several other articles have explored pre-whitening and its effects in detail.^47^^,^^67^ Pre-whitening greatly reduces the FDR in the connectivity models. Here, pre-whitening was implemented using an AR model as follows:

fNIRS time course can be expressed as

$$y_{t}=\overset{p}{\underset{i=1}{\sum }}a_{i}\cdot y_{t-i}+\varepsilon_{t}\;\forall \;t,$$

and $\varepsilon_{t}\in N(0,\sigma^{2})$.

Here, $y_{t}$ is the fNIRS time series, $a_{i}$ are the AR parameters, p is the model order, which is determined using Bayesian information criterion (BIC), and $\varepsilon_{t}$ is the innovation term. The innovation term is used to calculate the connectivity metrics rather than the time series themselves. Figure 4 shows the (a) power spectral density, and (b) autocorrelation function plots for a representative channel before and after pre-whitening for three cases of simulated data including, (i) with temporal autocorrelation, (ii) with temporal autocorrelation and global systemic physiology, and (iii) with temporal autocorrelation, global systemic physiology, and motion artifacts. As expected, the pre-whitening step flattens the power spectral density plot and reduces the temporal autocorrelation in the fNIRS time series.

**Fig. 4:**
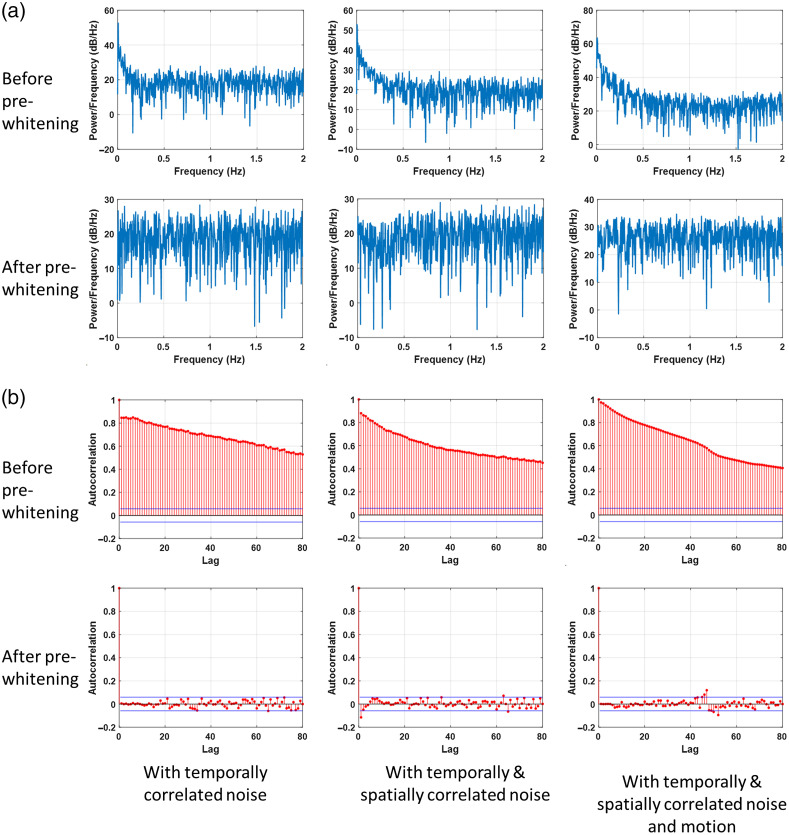
Plots showing the (a) power spectral density and (b) autocorrelation function plots for a representative channel before and after pre-whitening for three cases of simulated data in each column including, (i) with temporal autocorrelation, (ii) with temporal autocorrelation and global systemic physiology, and (iii) with temporal autocorrelation, global systemic physiology, and motion artifacts.

#### 2.3.2 Robust methods

After pre-whitening the time series, the innovation terms contain information added at that time point. The large signal changes due to motion are more prominent as they came from a different data generating process (motion) relative to the neural data generating process. Because motion artifacts are considered outliers, they can affect assumptions of normality. Often these high influential points can have a significant impact on the estimates of connectivity metrics. As mentioned earlier, motion artifacts affect connectivity metrics differently depending on whether the motion is shared across many channels or if it is limited to just a few channels. Motion corrupted timepoints that are shared across multiple channels can impact the functional connectivity by increasing the covariance between them. Thus, to identify statistical outliers, we used a multivariate version of a joint weighting function that was previously introduced in Santosa et al.’s work.^47^ The joint weighting function can identify the outliers by computing the geometric length of the time series and downweighting them appropriately depending on how they deviate from the mean, with motion corrupted outliers being assigned lower weights, which results in them having a lower impact on the correlation and regression methods.

A geometric length function $r_{t}$ is computed from the innovation terms of all the channels (including short-separation channels if available) as follows:

$$r_{t}=\sqrt{\varepsilon_{1,t}^{2}+\varepsilon_{2,t}^{2}+\varepsilon_{3,t}^{2}\ldots \ldots \varepsilon_{c,t}^{2}},$$

where $\varepsilon_{i,t}$ indicates the innovation terms of the i’th channel for timepoint t, and c is the total number of channels.

A weighting function (S) that downweights outliers depending on how much they deviate from the mean, can be calculated as follows for all timepoints using the square root of Tukey’s bisquare function

$$S(\frac{r}{\sigma })=1-{\left(\frac{r}{\sigma \cdot \kappa }\right)}^{2}\;\text{for}\;|\frac{r}{\sigma }|<\kappa \;0\;\text{for}\;|\frac{r}{\sigma }|>\kappa .$$

Here, σ is the standard deviation of the distribution and is estimated from the relationship between median absolute deviation (MAD) and the standard deviation of a normal distribution (σ≈1.4826*MAD.) MAD is used because it is a more robust dispersion statistic.^79^ The constant κ is set to a value of 4.685 which provides 95% efficiency for a standard normal distribution without any outliers. A weighting matrix S with diagonal elements representing the weighting function for all timepoints can be multiplied with the innovation terms for all the time series to downweight the statistical outlier points from the normal distribution

$$Y_{c}=S\cdot E_{c}.$$

Here $Y_{c}$ is the reweighted innovation terms matrix for all channels, and $E_{c}$ is the innovation terms obtained after pre-whitening.

So, to reduce the impact of motion artifacts, for regression models, instead of using a linear model and estimating the parameters with ordinary least squares estimates, we use robust statistics that use weighted least squares (WLS) to downweight the outliers calculated from the studentized residuals. The weights are calculated using Tukey’s bisquare function using the process detailed earlier. The weights calculated are then applied to the original data, and the residuals are re-estimated. The process is repeated till convergence. Robust regression is implemented in the in-built MATLAB function “robustfit.” The MATLAB function robustfit uses a convergence criterion of 50 iterations or no detectable numerical change in the values of the regression coefficients, whichever is earlier, to assess the convergence of the coefficients. Since multivariate Granger causality (MVGC) and modified MVGC did not converge in 50 iterations, we changed the maximum iteration limit to 150 iterations, to ensure that the regression parameters converged. The parameters estimated using the WLS regression can be obtained as

β^=(XTSX)−1XTSy.

Here, β^ is the parameter estimates and S is the weighting matrix.

More details about the use of robust statistics in fNIRS data processing can be found in other articles.^47^^,^^54^^,^^67^

#### 2.3.3 Principal components analysis-based regression for reducing global signal changes

If the probes are widely distributed across the brain, then the large signal variance shared across multiple channels may mostly reflect physiological noise.^51^ Hence, the first few components of PCA can be used to identify the global signal changes due to physiological noise and regress out its effects from the channel data. The data covariance matrix can be decomposed using singular value decomposition using

$$C=U\cdot \Sigma \cdot V^{T},$$

where C is the data covariance matrix given $C=Y^{T}\cdot Y$, U is a matrix containing left singular vectors. Σ is a diagonal matrix containing singular values and V is the matrix containing right singular vectors. Usually, the first few components explaining the most variance are used. We used the minimum number of principal components that explained at least 90% of the channel covariance. The global signal can be extracted from the decomposition of covariance of just the long-distance channels, just the short-separation channels, or both. All three options were examined in our testing. The channel data after PCA regression be obtained as

$$y_{\mathrm{PCA}\;\mathrm{filtered}}=[I-Z\cdot {\left(Z^{T}\cdot Z\right)}^{-1}\cdot Z^{T}]\cdot y.$$

Here, y is either the innovation term or the raw data for the channel depending on the analysis pipeline. Z is the matrix containing the first few components as columns.

### 2.4 Functional Connectivity Metrics

In this work, we examined several ways to estimate functional connectivity. Here, we focus only on methods in the time domain including correlation, partial correlation, and several variations of Granger causality. Time-frequency methods, such as wavelet coherence were not examined in this work for two reasons. First, in our previous work,^47^ we found that in bivariate connectivity analysis, wavelet coherence outperformed standard Pearson correlation in ROC analysis but did not perform as well as our pre-whitened, robust correlation models. Wavelet coherence also had high false positive rates and uncontrolled type-I errors unless the standard wavelet coherence model^80^^,^^81^ was modified to include higher-order AR terms and robust (outlier rejection) methods. With these modifications, we found no benefit to wavelet coherence over our pre-whitened robust models. Second, to our knowledge, a multivariate and/or partialed version of wavelet coherence has not been described and would require the development of a new statistical model.

#### 2.4.1 Correlation-based analysis models

*Pearson correlation coefficient*. Pearson correlation coefficient between two time series x and y is calculated as

$$r=\frac{\sigma_{x,y}}{\sigma_{x}\sigma_{y}},$$

where $\sigma_{x,y}$ is the covariance of time series x and y and $\sigma_{x}$ and $\sigma_{y}$ are the standard deviations of x and y, respectively. Standard Pearson correlation is not a robust statistical estimator and several robust variations have been proposed,^82^ and described in the context of fNIRS by Santosa et al.^47^ Robust correlation is calculated as the geometric mean of the standardized regression coefficients on the regression models y on x and x on y, to reduce outliers in either x or y, which can still influence the correlation estimates. The procedure is described below

$$y=[\begin{array}{cc} 1 & x \end{array}][\begin{array}{c} \beta_{0,1}\\ \beta_{x\to y} \end{array}].$$

A weighting matrix is estimated based on the studentized residuals in the above regression model that downweights the outliers in y. The weighting matrix $S_{x\to y}$ is then multiplied on both sides of the regression model above and the estimates of the parameters are updated,

$$S_{x\to y}.y=S_{x\to y}[\begin{array}{cc} 1 & x \end{array}][\begin{array}{c} \beta_{0,1}\\ \beta_{x\to y} \end{array}].$$

Similarly, we can fit x on y,

$$x=[\begin{array}{cc} 1 & y \end{array}][\begin{array}{c} \beta_{0,2}\\ \beta_{y\to x} \end{array}].$$

A weighting matrix $S_{y\to x}$ is then estimated based on the studentized residuals to downweight the outliers in x. $S_{y\to x}$ is multiplied on both sides of the regression model above and the estimates of the parameters are updated

$$S_{y\to x}.x=S_{y\to x}[\begin{array}{cc} 1 & y \end{array}][\begin{array}{c} \beta_{0,2}\\ \beta_{y\to x} \end{array}].$$

The two regression models are solved alternatively until the parameters $\beta_{y\to x}$ and $\beta_{x\to y}$ converge. The final robust correlation coefficient is estimated using the formula

$$\|r_{\text{robust}}\|=\sqrt{\beta_{x\to y}.\beta_{y\to x}}.$$

The sign of the robust correlation coefficient estimate is determined by the signs of the regression coefficients. The effective degrees of freedom given by $\sum (\min(S_{y\to x},S_{y\to x},S_{y,x}))-2$. The effective degrees of freedom are reduced due to the downweighting of the data points especially the outliers.

For both the standard and robust correlation estimates, the probability of observing the data given the null hypothesis (p-value) can be estimated from the T-statistic using

$$t=\frac{r\sqrt{df}}{\sqrt{1-r^{2}}},$$

with df is the effective degrees of freedom with a value of n−2 for standard Pearson correlation and r is the Pearson correlation coefficient. Here n is the number of time points.

*Autoregressive correlation*. The AR correlation was Pearson correlation coefficient calculated on the pre-whitened time series. As described in Santosa et al., a p’th order AR model is estimated for each channel of data and used to pre-whiten the signal.^47^ The innovations term in the AR model (e.g., the new information added at each time step) is then used in the correlation analysis. We used a maximum model order of 40 (10× the sample rate), which is higher than the AR filter used in simulating the fNIRS data. Based on empirical observations, the model order is typically $<10\cdot F_{s}$ (here $F_{s}$ is the sampling frequency) in experimental resting-state fNIRS data.

*AR Partial correlation*. Partial correlation involves a two-step process in which the other channels are first projected out of the data of interest and then the correlation is performed on the residuals. We used PCA regression-based projection to partial out the effect of other channels as described in Sec. 2.3.3. PCA regression is used to avoid collinearity instabilities of the model. The minimum number of eigenvectors to explain at least 90% of the spatial covariance was used. We examined the case where (1) only the other long-distance channels were used in the PCA, (2) only the short-separation channels were used, and (3) both long-distance and short-separation channels were used. For two channels i and j, if the innovation terms after pre-whitening are denoted by $\varepsilon_{i}$ and $\varepsilon_{j}$, the whitened PCA filtered time series are calculated as

$$\varepsilon_{i,\mathrm{PCA}\;\text{filtered}}=[I-Z\cdot {\left(Z^{T}\cdot Z\right)}^{-1}\cdot Z^{T}]\cdot \varepsilon_{i},\;\varepsilon_{j,\mathrm{PCA}\;\text{filtered}}=[I-Z\cdot {\left(Z^{T}\cdot Z\right)}^{-1}\cdot Z^{T}]\cdot \varepsilon_{j}.$$

Now, the AR partial correlation can be calculated as the Pearson correlation between the two residuals as the global signal is removed from the channels. The degrees of freedom are further reduced based on the number of principal components retained. The input data to this model was the pre-whitened innovations time series rather than channel data, hence this method is termed AR partial correlation.

#### 2.4.2 Granger causality-based analysis models

*Multivariate Granger causality*. MVGC tests for lagged relationships between two time series after controlling for the effects of other time series. This is essentially a model-fit test of two regression models. We can test whether x “Granger causes” y after controlling for the channels Z using multivariate AR models (MVAR) as detailed below. For a channel time series, we can model the current timepoint $y_{t}$ as a function of previous lags of y and other channels Z in the restricted model

$$y_{t}=\overset{p}{\underset{i=1}{\sum }}\alpha_{i}y_{t-i}+\overset{nPC}{\underset{j=1}{\sum }}\overset{p}{\underset{i=1}{\sum }}\gamma_{i}z_{j,t-i}+c_{1}+\varepsilon_{t}.$$

(restricted model)

In the unrestricted model, we add the lag terms of x, to model the current timepoint in y

$$y_{t}=\overset{p}{\underset{i=1}{\sum }}\alpha_{i}y_{t-i}+\overset{p}{\underset{i=1}{\sum }}\beta_{i}x_{t-i}+\overset{nPC}{\underset{j=1}{\sum }}\overset{p}{\underset{i=1}{\sum }}\gamma_{i}z_{j,t-i}+c_{2}+\varepsilon_{t}.$$

(unrestricted model)

Here, p is the number of lag terms included in the model, it is determined using fit measures using BIC. To test for causality, we statistically test if adding the additional lag terms in x (unrestricted model) improves the predictability of $y_{t}$ compared to the model without x (restricted model). If the past of the x contains predictive information about the current timepoint $y_{t}$, then the error in the prediction of $y_{t}$ is improved even after controlling for the lost degrees of freedom. This can be assessed statistically as

  Null hypothesis: x does not Granger cause y

$H_{0}$: $\beta_{1}=\beta_{2}=\beta_{3}=\ldots \beta_{p}=0$.

  Alternative hypothesis: x does Granger cause y.

$H_{A}$: at least one of $\beta_{i}$ is non-zero

Since we have nested models, we can use a nested F-statistic to test for Granger causality

$$F=\frac{{\mathrm{SSE}}_{R}-{\mathrm{SSE}}_{U}/p}{{\mathrm{SSE}}_{U}/[n-(2p+1)]}.$$

Here, ${\mathrm{SSE}}_{R}$ and ${\mathrm{SSE}}_{U}$ are the sums of squared errors of the restricted and unrestricted models respectively and n is the number of timepoints. So, we can calculate a p-value for the F-statistic at degrees of freedom p and [n−(2p+1)]. A Granger causality metric G, can also be calculated to assess the strength of the lagged relationship as follows:

$$G=\log\;{\mathrm{SSE}}_{R}/{\mathrm{SSE}}_{U}.$$

The purpose of using MVGC over bivariate Granger causality is to control for the effects of third variables including systemic physiology. The addition of lag terms of several channels in the MVGC model can easily make the regression model unsolvable as the number of variables would exceed the number of timepoints. So rather than using the channel data itself, we first perform PCA and included the fewest components that explained at least 90% of the variance as we did with partial correlation earlier. We then include components and the time-lagged components rather than the channel data in the restricted and unrestricted models.

Modified multivariate Granger causality. Traditional MVGC includes only the time-lagged history of y, x, and Z on the right-hand side of the equations. Thus, MVGC only models non-zero lagged relationships (hence the causality term). In comparison, the correlation methods described in Sec. 2.4.1 only model zeroth-lag correlation. It is important to recognize that “zeroth-lag” is relative to the sample rate and that at a 4-Hz sampling of the simulated fNIRS data, any synchronous signal changes occurring within one sample (250 ms) would be mathematically zeroth-lag. While neural signaling in the brain is expected to be fairly fast (100 s of milliseconds), we did not want to assume that all correlations would be zeroth-lag, and in this paper, we examined multiple scenarios. Here, we introduce a modified MVGC model to model both lagged and zero-lagged relationships between channels. In the modified MVGC we include the zero-lag terms of Z (top principal components) in both the restricted and unrestricted model as well as the zero-lag terms of channels x in the unrestricted model. So, the restricted model can be written as

$$y_{t}=\overset{p}{\underset{i=1}{\sum }}\alpha_{i}y_{t-i}+\overset{nPC}{\underset{j=1}{\sum }}\overset{p}{\underset{i=0}{\sum }}\gamma_{i}z_{j,t-i}+c_{1}+\varepsilon_{t}.$$

(restricted model)

Similarly, the unrestricted model can be written as

$$y_{t}=\overset{p}{\underset{i=1}{\sum }}\alpha_{i}y_{t-i}+\overset{p}{\underset{i=0}{\sum }}\beta_{i}x_{t-i}+\overset{nPC}{\underset{j=1}{\sum }}\overset{p}{\underset{i=0}{\sum }}\gamma_{i}z_{j,t-i}+c_{2}+\varepsilon_{t}.$$

(unrestricted model)

The aforementioned equations also include the zero-lagged terms of Z and x. Now, to test for statistical significance of Granger causality, we test a null hypothesis and alternative hypothesis as follows:

  $H_{0}$: $\beta_{0}=\beta_{1}=\beta_{2}=\beta_{3}=\ldots \beta_{p}=0$.

  $H_{A}$: At least one of $\beta_{i}$ is non-zero

The statistical significance is assessed with the nested F-statistic and the Granger causality is calculated similarly to traditional MVGC. One caveat of this modified model is that the hypothesis does not test whether the connection between y and x was due to the zeroth or non-zeroth lag terms. One could examine this further by modifying the unrestricted model to also contain the non-zero lagged terms of x, such that the only difference between the unrestricted and restricted models is the inclusion of the zeroth-lag term in x.

*Robust multivariate Granger causality*. Finally, we developed a robust statistical version of both the MVGC and modified MVGC models. The definition of outliers in this multivariate model is a bit challenging since, such as the bivariate correlation model, outliers can exist in the time courses of y, x, and/or Z. In bivariate correlation, we used the union of outlier weights (e.g., an outlier in y or x). However, we found that the extension to many channels of data often resulted in too much of the data being downweighted since an outlier point in any one of the channels caused that time point to be downweighted for all the channels. In particular, the time lags in the MVGC required all lagged columns of the matrix containing that entry to be downweighted as well.

To reduce the complexity of robust regression methods described earlier with multiple terms (channels and their lag terms) in the regression model, rather than using channel data by itself, we use the innovation terms. The procedure for robust MVGC is described below. First, the channel data (both short-separation and/or long-distance) are pre-whitened and the innovation terms are calculated. Data are pre-weighted with a weighting matrix calculated from the innovation terms in a procedure described in Sec. 2.3.2. After pre-whitening and pre-weighting, the innovation terms of the other channels (Z) is included in the regression for the restricted model, described as follows:

$$\varepsilon_{t}^{y}=\overset{nPC}{\underset{j=1}{\sum }}\overset{p}{\underset{i=0}{\sum }}\gamma_{i}\varepsilon_{j,t-i}^{z}+c_{1}+\varepsilon_{t}$$

(restricted model).

Here, $\varepsilon_{t}^{y}$ is the innovation term of channel y at timepoint t after pre-whitening and pre-weighting. $\varepsilon_{j,t-i}^{z}$ are the principal component weights for the j’th component at timepoint t-i. *nPC* denotes the number of principal components used.

Similarly, the innovation terms are also used in the unrestricted model rather than the channel data itself:

$$\varepsilon_{t}^{y}=\overset{p}{\underset{i=1}{\sum }}\beta_{i}\varepsilon_{t-i}^{x}+\overset{nPC}{\underset{j=1}{\sum }}\overset{p}{\underset{i=0}{\sum }}\gamma_{i}\varepsilon_{j,t-i}^{z}+c_{2}+\varepsilon_{t}$$

(unrestricted model).

Compared to the restricted model, the unrestricted model additionally includes the innovation terms and lags of channel x. $\varepsilon_{t-i}^{x}$ is the innovation term of channel x at timepoint t-i.

A weighted least-square regression using an iterative robust estimator is used to fit both the restricted and the unrestricted models with the data being downweighted from the estimated studentized residuals. The process is repeated until convergence of the weight matrix and parameter estimates. As with other MVGC models, nested F-statistic is used to test for the statistical significance of the lagged relationship between the two time series.

### 2.5 Data Analysis Plan

To test the effectiveness of each processing method in reducing the effects of temporal autocorrelation, global shared physiology, and motion artifacts, all the connectivity metrics were compared. We expected that all methods which had the pre-whitening step would correct for temporal autocorrelation. To test the need for correcting for global signals due to shared physiological noise between the channels, we expected methods that controlled for the effects of other channels such as partial correlation and MVGC would perform better when the data had systemic physiology. We also expected the robust methods to perform better than the non-robust methods when the simulated data had added motion artifacts. Finally, we expected the zero-lag correlation measures to perform better when the shared simulated neural covariance was at zero-lag and the Granger causality methods to perform better when the information was present in the first lag. The modified Granger causality with zero-lag which models both zero-lag and lagged relationships is expected to perform better at both the zeroth-lag and the first lag. In Fig. 5, we provide a summary of the different analysis pipelines examined.

**Fig. 5:**
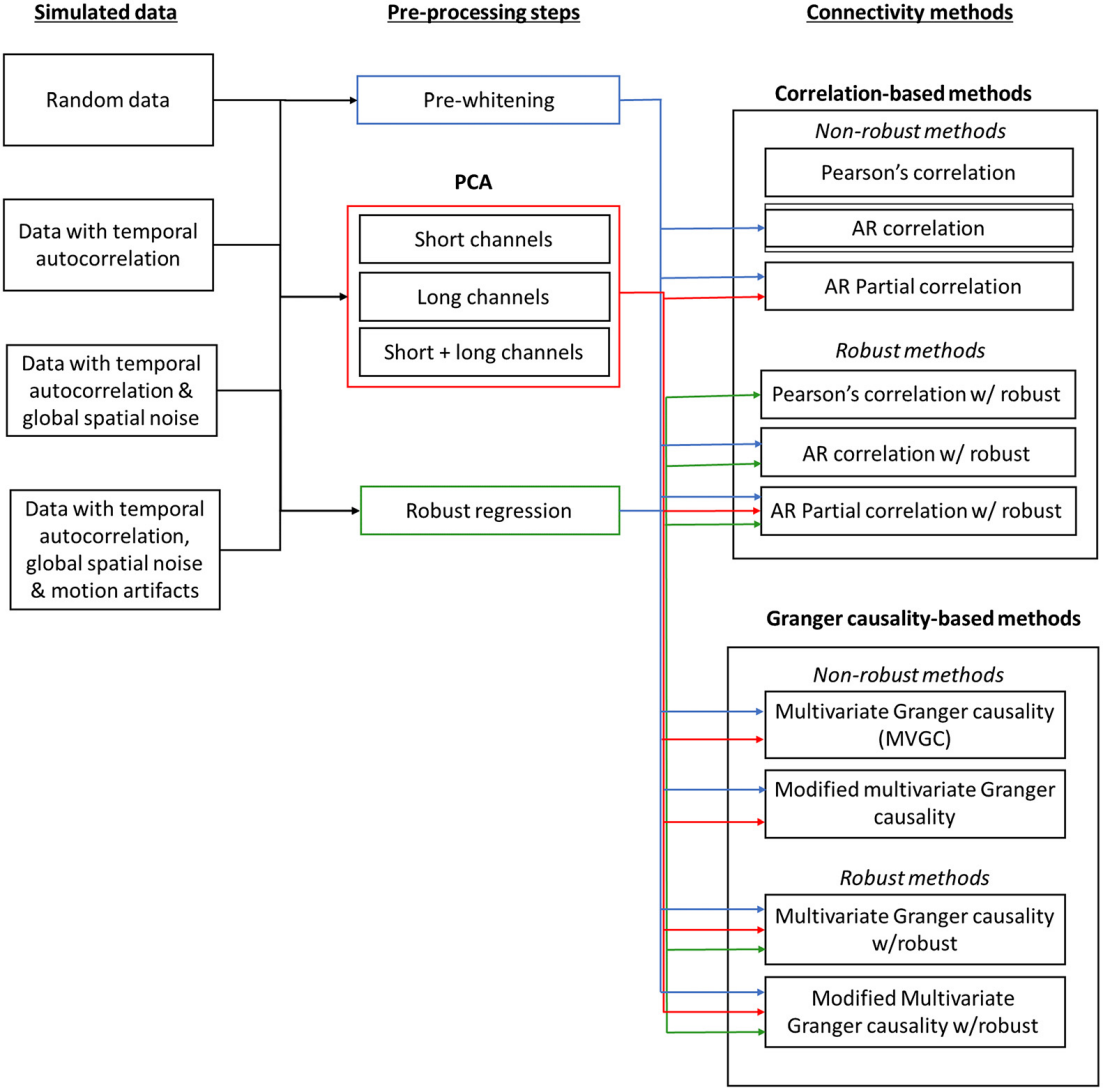
Figure showing different pipelines and connectivity methods that were applied to the four types of simulated data.

We also compared the effectiveness of robust versions of (1) Pearson correlation, (2) AR correlation, and (3) AR partial correlation on the experimental resting-state fNIRS data from 24 subjects. After group-level analysis, with subject as a random factor, we expected AR partial correlation to show the sparsest network, followed by AR correlation. We expected the Pearson correlation to show the densest network with almost every channel connected with the other. Since we do not know the ground truth nor do we have a “gold standard” such as fMRI to compare our results to, we cannot draw broad conclusions about performance of these analyses methods.

### 2.6 Performance Metrics

We ran the simulation 200 times, each time generating a different dataset with different covariance structure to mimic shared neural activity. Then, we calculated the p-values from the ‘correlation coefficient’ for correlation-based methods or an F-statistic for Granger causality methods. The false positives, true positives (TPs), true negatives (TNs), and false negatives were tallied up to compare the effectiveness of each of the multiple data processing methods for quantifying connectivity. We used two plots to assess the performance of the different methods (1) type-I error control plots and (2) ROC curves.

#### 2.6.1 Type-I error control plots

To understand how well the data processing methods control for type-I error, we plotted the actual false positive rate (from bootstrapping) against the theoretical p-value obtained from the test statistic for the method (denoted by p-hat). An ideal method would indicate that the p-value from the method (p-hat) would match the actual p-value, hence the plot would have a unit slope. Parts of the curve above the unit slope line, indicate the model is likely to have more false positives than expected at that significance level, and the parts under the line indicate that the model is likely to have more false negatives than expected at that significance level.

#### 2.6.2 ROC curves

To investigate the sensitivity and specificity of the data processing methods and the model performance, ROC curves were generated from the known ground truth as some channels were simulated with shared “neural” covariance. The ROC curve plots the TP rate (sensitivity) versus false positive rate (1-specificity) at different thresholds. Ideal performance of the processing method would result in very high TPs and very high TNs approaching the top left corner of the plot. Similarly, a curve with slope one and an area-under-the-curve (AUC) of 0.5 would indicate random guessing. Since the number of TPs was less than the TNs (only 10% of the non-diagonal elements in the covariance matrix used to generate the simulated data were non-zero) in the simulated data, a random subsample of TNs equal in size to the TPs was used to generate the ROC curves.

### 2.7 Code Implementation

All of the methods were implemented in MATLAB (The MathWorks, Inc., Natick, Massachusetts). Inbuilt MATLAB functions and the functionality of the NIRS Brain AnalyzIR toolbox^75^ were used for preprocessing and implementation of the correlation methods. Other methods including partial correlation, Granger causality, and robust Granger causality were implemented using custom MATLAB scripts. The toolbox and the codes used for this article are available online at https://github.com/pradlanka/rsfc-fnirs or can be obtained by request from the corresponding author.

## 3 Results

### 3.1 Comparison of Processing Methods

As expected, as the progression of simulations and challenges of the noise increased, the basic models such as standard Pearson correlation quickly failed. For example, Pearson correlation assumes independent measurements, which is violated by the addition of temporally autocorrelated noise. As shown in Fig. 6(b), the Pearson and robust Pearson correlation models which did not include an AR (pre-whitening) filter had considerably lower performance on ROC curves compared to the pre-whitened and MVGC models. Almost all other methods have an AUC close to 1. In addition, as shown in Fig. 6(a), the Pearson and robust-Pearson models had substantial false positive rates as indicated by lines above the diagonal in the plot of actual false positive rate versus expected rate (p-hat). For example, at an expected p-hat of 0.05, the actual false positive rate was over 80%. The other analysis models had type-I error plots much closer to ideal (diagonal) where the expected rate equals the realized rate. The AR-correlation and robust AR-correlation both had the closest to ideal type-I error control. The AR partial correlation model had a slight overestimation of the significance of the test statistic, whereas the robust AR partial correlation overcorrects leading to underestimation of significance of the test statistic (less than expected false positive rate). Both versions of the modified MVGC tended to underestimate the p-values slightly, thus leading to slightly higher than expected false-positive rate. Here, we are not showing the results for the standard MVGC model, which does not include a specific zeroth-lag term. Because the data were simulated at the zeroth-lag, which the MVGC does not model, the MVGC shows no significant channels. These results support the necessity of correcting for the autocorrelation in the time series as highlighted in a previous article.^47^

**Fig. 6:**
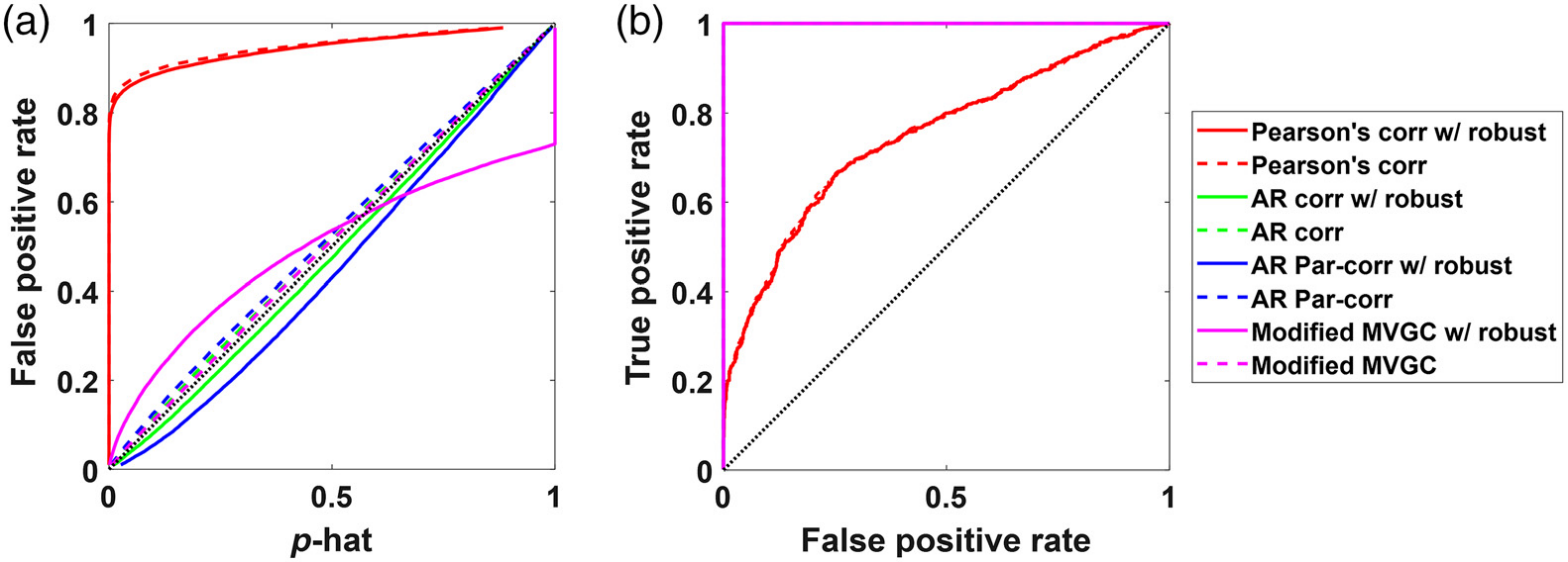
The (a) type-I error control plots and (b) ROC curves for all the methods with simulated data containing temporal autocorrelation. The dotted lines indicate the nonrobust version of the methods, while the solid lines represent their robust counterparts.

The second set of simulations (Fig. 7) was generated using both temporal autocorrelation and spatially global systemic physiology. As expected, AR correlation joins the Pearson correlation coefficient in increased false positives [Fig. 7(a)] and lower AUC in the ROC curves [Fig. 7(b)]. While the use of pairwise correlation worked when the noise was independent between channels, spatial noise introduced global false positive connections and large over-reporting of the significance of the model. AR Partial correlation and the modified (zero-lagged) Granger causality still performed well. Again, all the analysis methods except pairwise Pearson correlation had AUC of near 1 on the ROC curves, which indicates that the TP channels were still well separated from the negatives, but that the reporting of the p-values (p-hat) was very wrong. In other words, one can find the true connections by setting the right threshold on the correlation coefficient, but that one cannot trust the reported p-value for setting that threshold. In the results for both the simulations shown in Figs. 6 and 7, there were no motion artifacts added to the simulated data and thus the robust and non-robust methods performed similarly well with their type-I error control curves and the ROC curves. Though the use of robust methods can lead to a loss in degrees of freedom as the weights of data points can be <1, the number of timepoints is still much larger (>1000), hence the loss in degrees of freedom is almost negligible.

**Fig. 7:**
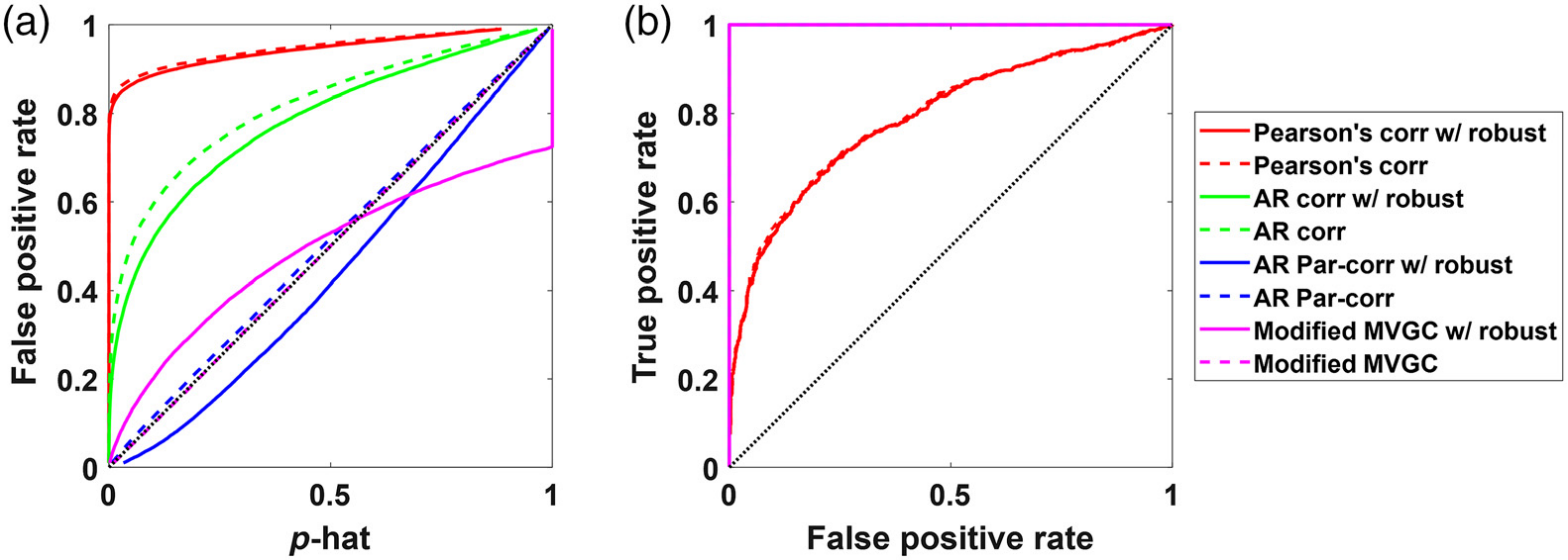
The (a) type-I error control plots and (b) ROC curves for all the methods with simulated data containing temporal autocorrelation and shared global signal mimicking systemic physiology. The dotted lines indicate the nonrobust version of the methods, while the solid lines are their robust counterparts.

For, the third simulation, head motion modeled as spike and shift artifacts was added to the simulated data containing both the temporal autocorrelation and global signal. As shown in Fig. 8, the robust methods which downweights outlier points, performed much better than their non-robust counterparts, with robust modified Granger causality and AR partial correlation with short-separation channels having similar AUC, but AR partial correlation had fewer false positives than modified MVGC. It is important to note that while robust modified Granger causality was effective at reducing the false positives it still had much higher-than-expected false positives (false positive rate of 0.25 at an expected rate of 0.05).

**Fig. 8:**
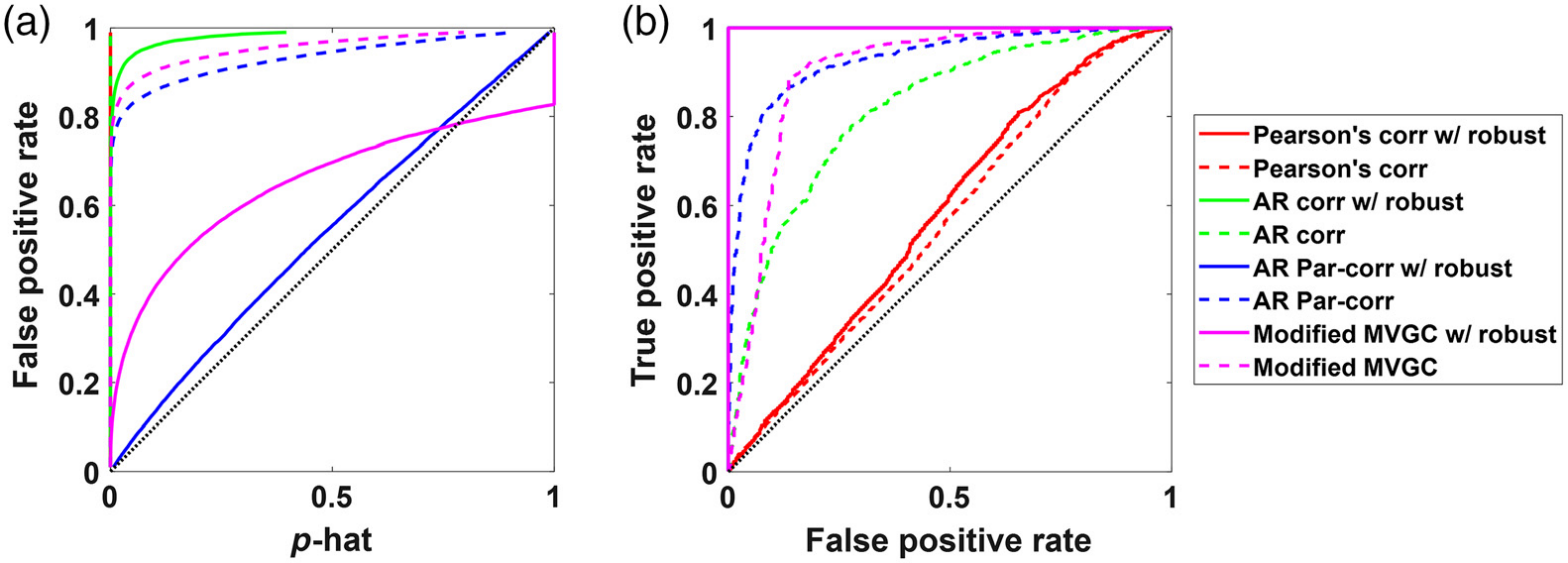
The (a) type-I error control plots and (b) ROC curves for all the methods with simulated data containing temporal autocorrelation, shared global signal mimicking systemic physiology and head motion artifacts. The dotted lines indicate the nonrobust version of the methods, while the solid lines are their robust counterparts.

### 3.2 Comparison of the Effectiveness of Short-Separation Channels

In the previous section, we showed AR partial correlation and modified MVGC models that included both the short-separation and other long-distance fNIRS channels in the model. Our results indicated that using these components was important to remove global noise signals. In this section, we explore these models in more depth and compare the use of just short-separation channels, the other long-distance channels, or both short-separation channels and other long-distance channels in the performance of the whitened partial correlation method. In Fig. 9(a), we show these comparisons for data simulated with both temporal and spatial noise. In the type-I error control plot, the use of only long-distance channels was the worst and likely led to the overestimation of significance of the test statistic (correlation coefficient) indicating that the results still retained too many global connections. The use of only short-separation channels produced the best result, although it slightly under-estimated the significance of the robust estimator and result in a slight introduction of false negatives in the estimates. Finally, the use of both the short-separation and long-distance channels was slightly worse than the use of the short-separation channels alone, but better than the use of only the long-distance channels. This is probably due to some overfitting in the model.

**Fig. 9:**
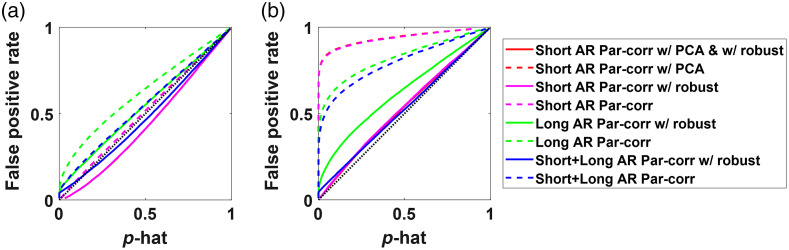
Plots showing type-I error control plots comparing whitened partial correlation methods using short-separation, long-distance channels or both on simulated data containing (a) temporal autocorrelation and shared global signal mimicking systemic physiology, and (b) temporal autocorrelation, systemic physiology, and added head motion. Results are shown for short-separation channels both with PCA and without PCA performed on them before partialing out its effects. The dotted lines indicate nonrobust versions of the methods, while the solid lines indicate their robust counterparts.

In Fig. 9(b), we show the comparisons for data simulated with additional motion artifacts. As expected, the robust statistical estimators greatly outperform the non-robust ones, with the short-separation channels-only model being the most sensitive to these artifacts. Consistent with the motionless simulations, the robust short-separation channels-only and robust version of both short-separation and long-distance gave the best results. The use of only long-distance channels in the robust model still had a substantial over-reporting of the significance of test statistics.

Finally, in Fig. 10, we examined the same analysis using the modified MVGC model in place of the AR partial correlation. Figure 10(a) shows the results for motion-less simulated data. We found that the MVGC was less sensitive to which channels were used in the model and the short-separation, long-distance, and both models all had similar performance. The robust version of the MVGC was observed to have a substantial over-reporting of the significance of the test statistics even with the motionless data simulations. We believe that this is an uncorrected error in the model concerning the effective degrees of freedom of the estimates. In particular, the definition of statistical outliers in the robust estimator is difficult because the time points in the measurement variables y, x, and/or Z can all be outliers due to motion. The Tukey’s bisquare weight being used in this model assumes the outliers are independent, but our current model does not effectively account for shared outlier time points across multiple channels and thus the degrees of freedom are slightly overestimated. In Fig. 10(b), the MVGC models are compared for the data simulations with added motion artifacts. Here, we found that robust methods out-perform their non-robust versions, but we again found over-reporting in the statistical estimates.

**Fig. 10:**
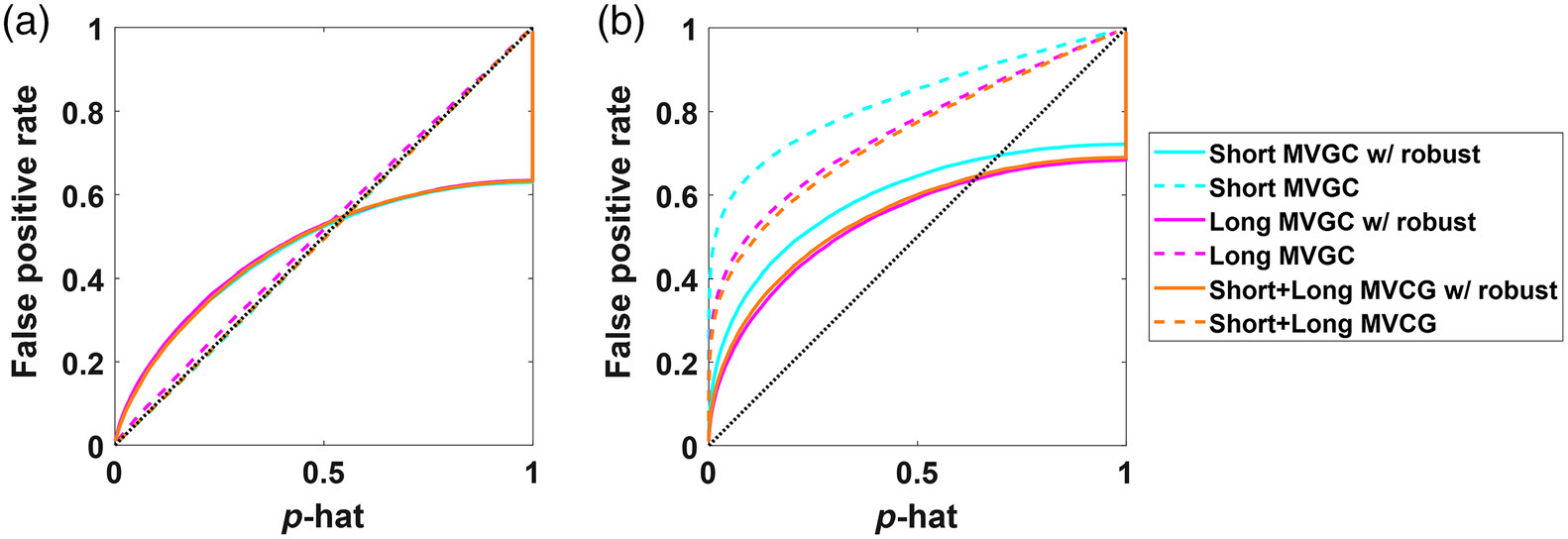
Plots showing type-I error control plots comparing MVGC methods using short-separation, long-distance channels, or both on simulated data containing (a) temporal autocorrelation and shared global signal mimicking systemic physiology, and (b) temporal autocorrelation, systemic physiology and added head motion. The dotted lines indicate the nonrobust version of the methods, while the solid lines are their robust counterparts.

### 3.3 Modeling Lagged Connectivity

So far, we have shown the results for the simulated data when the relationships between channels were present at the zero lag. The Pearson and partial correlation models all assumed zeroth-lag connections whereas the standard MVGC assumed only non-zero lag (causality). The modified MVGC considered both zero and non-zero connections. Thus, when the data were simulated with only zeroth-lag connections, the AR partial correlation was determined to be the best. Standard MVGC (with no zeroth-lag) was not even presented in the previous sections since it failed to find anything in the zeroth-lag simulations [Fig. 11(a)]. Likewise, when data were only simulated with relationships at non-zero lag terms, the AR partial correlation models failed, as shown in Fig. 11(b). The modified MVGC was the only model that worked in both cases.

**Fig. 11:**
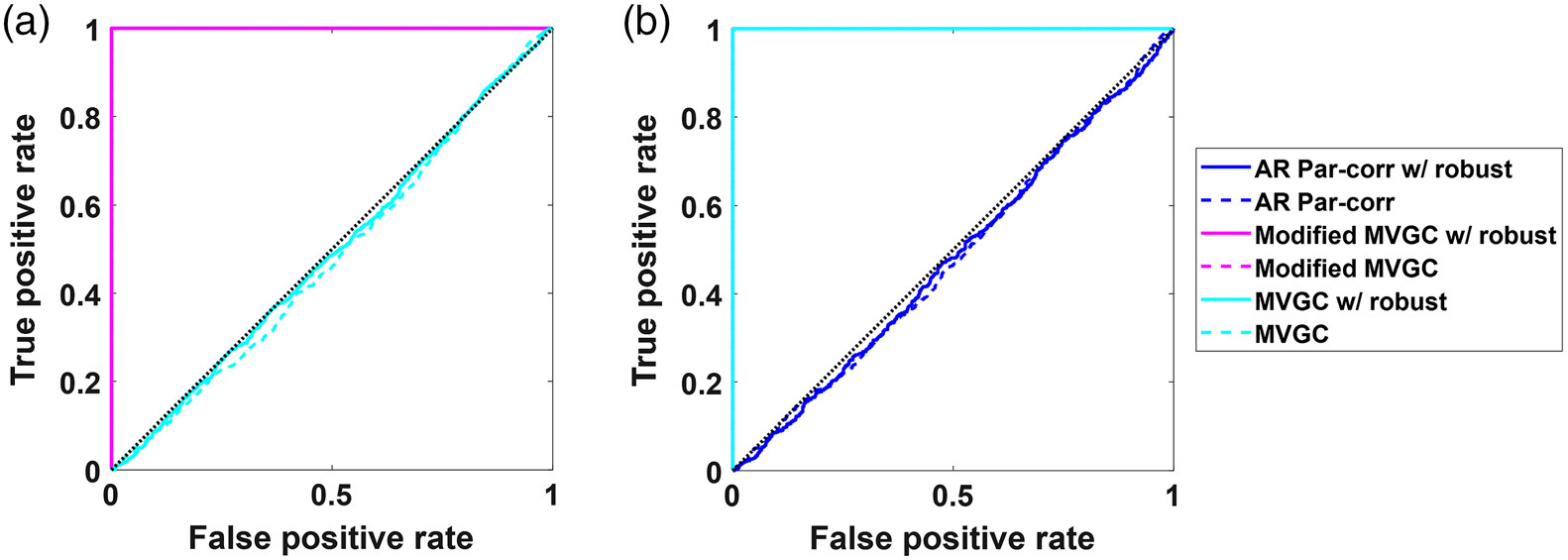
ROC curves comparing whitened partial correlation, MVGC, modified MVGC with and without robust regression when the linear relationships between channels are simulated at (a) zero-lag and (b) first lag. The simulated data contain temporal autocorrelation and shared global signal mimicking systemic physiology. The dotted lines indicate the nonrobust version of the methods, while the solid lines are their robust counterparts.

In Fig. 12, with motion artifacts added, the same pattern is shown in that the ROC curves are best for the robust AR partial correlation and robust modified MVGC models in the case of zeroth-lag simulations and best for robust MVGC and robust modified MVGC with non-zero lag simulations. In both cases, the modified-MVGC was not quite as good as the AR partial correlation or the MVGC, so if one knew what kind of connections were present, the modified-MVGC is not the preferred choice. However, in the absence of this knowledge, this was the only model to work in both scenarios.

**Fig. 12:**
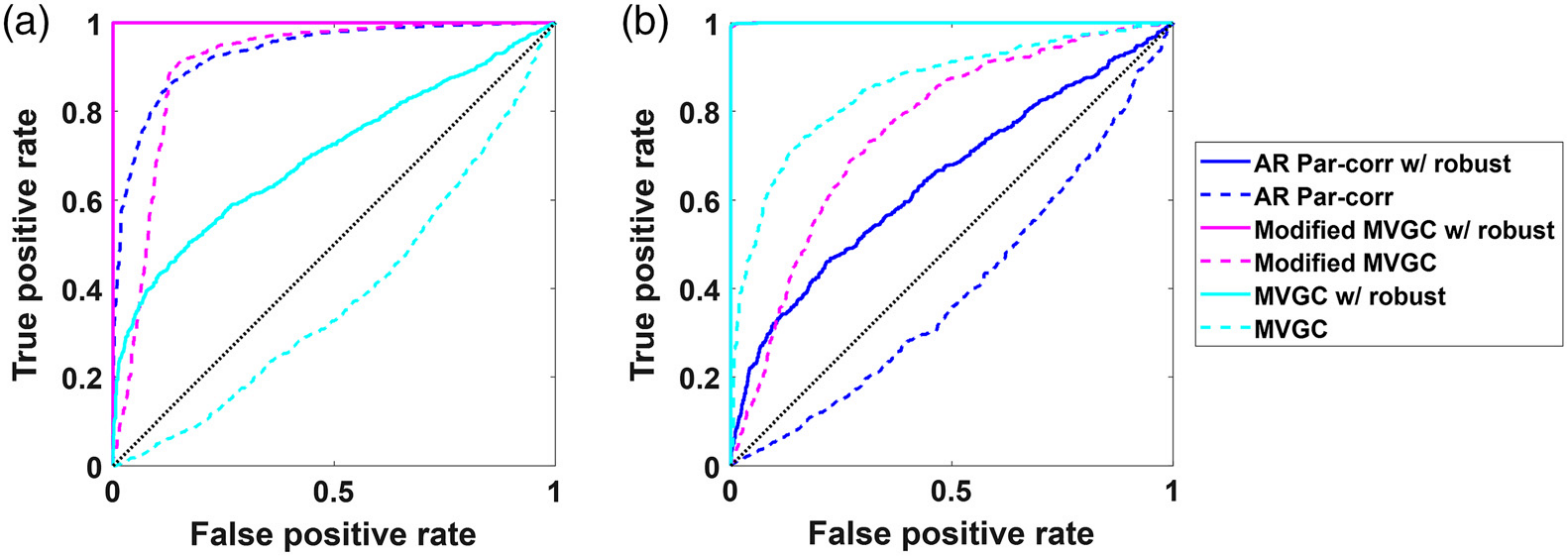
ROC curves comparing whitened partial correlation, MVGC, modified MVGC with and without robust regression when the linear relationships between channels are simulated at (a) zero-lag and (b) first lag. The simulated data contain temporal autocorrelation, shared global signal mimicking systemic physiology, and head motion. The dotted lines indicate the nonrobust version of the methods, while the solid lines are their robust counterparts.

### 3.4 Overall Simulation Results

Figure 13 shows the resulting null distributions produced by each of the simulation types and analysis methods. The dashed red line shows the theoretical distribution assumed by the statistical model. The closer the match between the empirical distribution and the theoretical one, the more accurate the reported statistics and the less bias in the estimates. We see that the standard Pearson correlation model was quite inaccurate for data with temporally correlated noise. The distribution was too wide compared to the theoretical, which means that p-values would be underestimated. Moving to the AR whitened correlation model (second column), we see that this distribution was fixed for the temporally correlated noise (no bias in the estimate), but in the presence of spatially correlated noise (middle row) was shifted from mean zero (bias in the estimate) and the distribution was too wide. The AR partial correlation model did correct for most of the global spatial noise, but then failed when motion artifacts were present. The robust AR partial correlation worked best under these conditions (bottom/right plot), although that distribution is still not ideal.

**Fig. 13:**
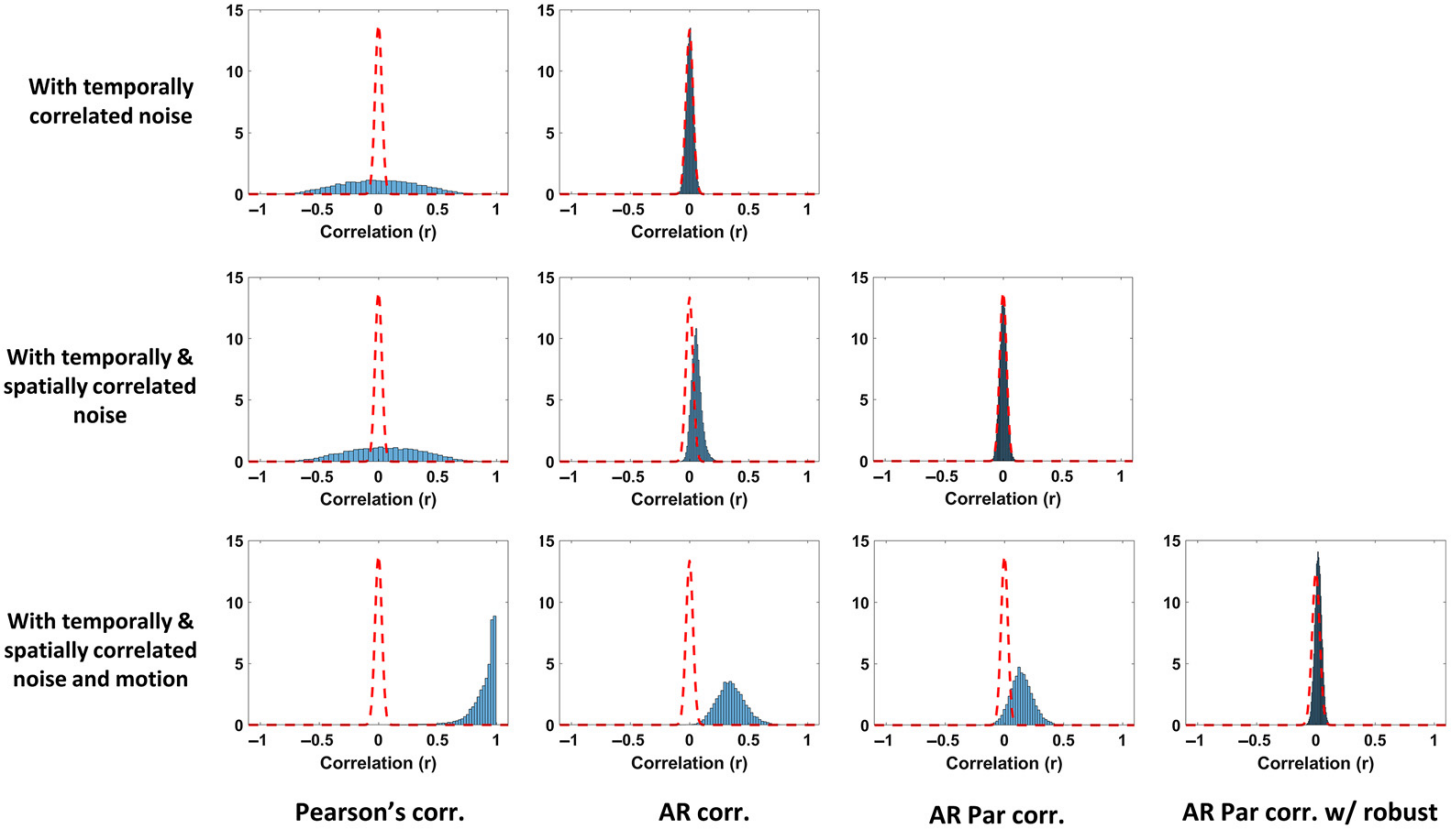
Plots showing the distribution of strength of the relationship between channels (correlation coefficient) quantified by the Pearson correlation, AR correlation and AR partial correlation, and robust AR partial correlation. The simulated data contain either temporal autocorrelation (first row), temporal correlation with spatial correlation due to systemic physiology (second row), and temporal correlation, spatial correlation, and head motion artifacts (third row). The dashed line indicates the expected distribution without physiological noise or head motion while the histogram shows the distribution of the actual correlation for each combination of simulated data and connectivity method.

### 3.5 Results on Experimental Resting-State fNIRS Data

To test the applicability of our functional connectivity methods on experimental resting-state data, we compared using robust versions of Pearson correlation, AR correlation, and AR partial correlation to model the functional connectivity between the channels on a sample of 24 participants. The group-level results with the significant paths (q<0.001, FDR correction) for the three methods are shown in Fig. 14. As the results indicate using AR partial correlation leads to much sparser networks, compared to AR correlation and Pearson correlation, as it corrects for global physiology and autocorrelation. Using a Pearson correlation revealed a very dense network of connections with most connections likely being false positives. Though, there is no known ground truth or gold standard RS-fMRI networks available for quantitative comparison of the methods, AR partial correlation would probably provide the lowest false positive rate among the three methods.

**Fig. 14:**
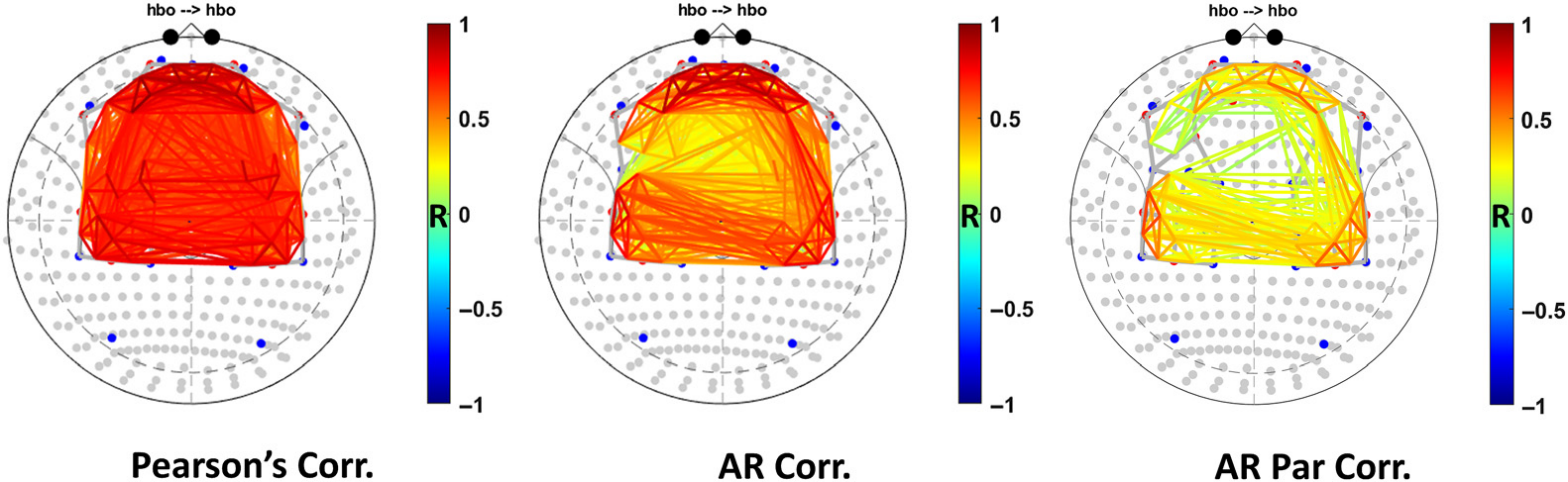
Comparison of the effectiveness of robust versions Pearson correlation, AR correlation, and AR partial correlation in correcting for temporal autocorrelation, global systemic physiology, and motion artifacts in experimental fNIRS data. The maps show significant (q<0.001, FDR corrected) connections between the channels projected on to the 10–5 coordinate system after group analysis using different connectivity methods based on changes in HbO.

## 4 Discussion

In the current article, we used simulated data to explore how physiological noise and the sluggish hemodynamic response together lead to temporal autocorrelation and spatial covariance in channel data, which in turn lead to increased false positives and lower AUC for the ROC curves. Although pre-whitening can reduce temporal autocorrelation, spatial covariance due to shared systemic physiology should still be corrected. Our results confirm and expand on previous studies by showing that a combination of pre-whitening with an AR filter and partial correlation of short-separation channel data to control for spatial covariance were effective in substantially lowering the false positive rate for resting-state connectivity analysis.

In particular, a shared systemic physiological signal can be captured from the short-separation or both short-separation and long-distance channels, using the first few principal components. We found that long-distance channels alone, while better than no corrections, were not sufficient to remove the global signals. The use of robust regression was modestly successful in controlling for signal changes due to head motion, especially for Granger causality, although it was very effective for partial correlation. One of the challenges in the multivariate models is regarding the definition of motion outliers. One way to consider a time point to be an outlier is if it is an outlier in any channel, which tends to lead to over-correction with many channels and when lagged terms are included (since all lags are also outliers). Another way is to consider a time point as an outlier when it is an outlier in a large enough subset of channels. We followed the latter approach using the geometric length of the innovations vector (across channels).

In this study, we also introduced a modified MVGC which included both the traditional lagged (causal) terms and a zeroth-lag component. This new model did relieve the assumptions about the zero or non-zero nature of the lag in connectivity and could be used in both scenarios. However, this model scored worse on the ROC analysis for specifically non-zero lag connections compared to the MVGC model and was more sensitive to motion (outliers) compared to AR partial correlation. For both the modified MVGC and MVGC, our robust statistical models were not perfect, and we found that both tended to over-report significance. This as mentioned earlier, may be due to the deficiency of the proposed methods in not fully accounting for the degrees of freedom in the model associated with the Tukey bisquare weighting of outliers in multiple channels of data simultaneously.

### 4.1 Use of Pre-Whitening Models

Temporal autocorrelation in fNIRS and fMRI time series is a known issue that leads to reduced degrees of freedom, increased variance of the correlation coefficient (not accounting causes underestimation of the variance), and thus increased false positives. Although pre-whitening has been suggested to reduce autocorrelation in time series and subsequently false discoveries in both fMRI^48^^,^^83^ and fNIRS,^47^ it must be noted that the use of pre-whitening as a processing step in resting-state analyses is not universally accepted. There are some concerns regarding pre-whitening the time series and its ability to distort the power spectrum which inherently follows the inverse power law.^53^^,^^84^ Blanco and colleagues argued that if the intrinsic signal is inherently colored, the use of pre-whitening may be inappropriate.^53^ These researchers observed that the anticorrelation between HbR and HbO signals is diminished by incorporating pre-whitening step in RS-fNIRS processing pipeline.^53^ However, it must be noted that although the HbO and HbR signals are in antiphase given the cerebral hemodynamics, their peaks are not maximally anticorrelated at zero-lag. Specifically, the positive peak of the HbO and the negative peak in HbR signals do not occur at the same time.^85^ Zeroth-lag is defined as within the same sample time, which means that this definition depends on the sample rate. As we see from the results in Figs. 11 and 12, when there is an intrinsic temporal lag in the underlying connectivity, correlation methods are not designed to capture this. Strictly speaking, HbO and HbR are not actually truly correlated, so much as they are causal in the mathematical sense with HbO preceding changes in HbR. Thus, the modified MVGC and MVGC methods are required to model these lagged signals and the finding that HbO and HbR are no longer correlated once autocorrelation is accounted for, is actually the expected mathematical result.

Recently, other methods than pre-whitening, have been proposed to control for the false positive rate in fMRI. These methods either correct for the reduced degrees of freedom or directly correct for the increased variance of the correlation. Some of these methods were compared on their effectiveness in accounting for the effects of autocorrelation in the time series on functional connectivity in fMRI.^84^ However, since fNIRS has a higher sampling rate than fMRI and therefore serial correlations are most prominent, the applicability and the utility of both pre-whitening methods and other methods need to be systematically compared for fNIRS datasets. Our findings differ from previous results from RS-fMRI. Unlike Arbabshirani et al.,^50^ who observed some bias in sample correlation coefficient (when the autocorrelation structures were different between the time series), our results were similar to Afyouni et al.,^84^ in not finding a bias in the estimate of the sample correlation coefficient for a zero correlation coefficient. Second, unlike the results of Arbabshirani et al.,^50^ wherein the bias in the estimate canceled out the inflated variance due to autocorrelation, our results show that the inflated variance of sample correlation due to autocorrelation in the time series was much higher. This is probably attributable to temporal dependencies at larger lags due to higher sampling rate of fNIRS data. Hence, correction for autocorrelation may be more important for fNIRS than for fMRI, and some of the conclusions from RS-fMRI may not be directly applicable to RS-fNIRS. It should also be noted that the method for correcting for autocorrelations can have important consequences for other steps in the preprocessing pipeline such as motion correction and reduction of spatial covariance using partial correlation. Furthermore, since the autocorrelation in the fNIRS time series is due to a combination of factors including, the hemodynamic response, high sampling rate, and presence of physiological noise, removing modeled physiological noise using external measurements, or short-separation filtering, though may reduce the need for higher AR model orders, the residual data are still autocorrelated as has been shown with short repetition times in fMRI.^86^ Further validation studies are necessary to support incorporating pre-whitening in the fNIRS data processing pipeline.

### 4.2 Use of Filtering Methods in the Preprocessing Pipeline

Filtering methods especially bandpass filtering are often used in the preprocessing pipeline for RSFC as it is assumed that filtering increases the signal-to-noise ratio by reducing the contributions of frequency bands with physiological and other noise sources. However, incorporating both filtering and pre-whitening in the preprocessing pipeline requires some careful thought. First, filtering—especially low-pass filtering—often increases the autocorrelation in the time series, worsening the issue of increased false positives in the data. So, any low-pass filtering needs to be performed before pre-whitening.^47^ Second, filtering removes the high-frequency components in the data, and since pre-whitening tries to whiten the frequency spectrum, it can make the estimates of the AR parameters during pre-whitening unstable. So any filtering should be done within the GLM model.^47^^,^^83^ Third, any filtering needs to be applied to both the signal and the noise model (including any physiological signals, short-separation channels) to prevent the reintroduction of the unwanted frequencies.^83^ Finally, any filtering applied to the data must account for the lost degrees of freedom from filtering, so that the significance of the correlations is not inflated along with increased false positives.^83^^,^^87^ In the current work, we did not incorporate filtering into the processing pipeline. Future studies must examine if indeed there is an increased sensitivity to the underlying neural activity by incorporating filtering into the preprocessing pipeline, despite some of the issues outlined above.

### 4.3 Use of Robust Methods to Reduce the Influence of Head Motion

Head motion artifacts in fNIRS are often statistical outliers as these changes are much larger in magnitude than the underlying physiological and intrinsic noise in the signal. The introduction of robust statistical estimators is required to avoid the over-leverage of these outlier points on the connectivity estimates. These approaches worked well for the correlation and partial correlation models but only offered a modest improvement for the MVGC models. In particular, these robust methods failed to converge at max iterations of 50 (but did converge when max no. of iterations was increased to 150), especially with MVGC, thus, limiting the use of robust MVGC for heavily motion-corrupted data. Future methods should probably explore estimating reliable and robust estimates of MVGC in presence of large motion artifacts. The issue with marking and correcting for head motion with MVGC is that a motion corrupted timepoint is downweighted across all lags, hence, there is a large reduction in the degrees of freedom when robust regression is used. Some studies correct for motion artifacts before the estimation of functional connectivity. These methods should explicitly control for the lost degrees of freedom due to motion correction in their analysis of functional connectivity. A related issue to the use of robust methods is to determine what timepoints are considered outliers and subsequently marked as motion artifacts. Aggressively labeling data as motion-related outliers, could reduce the degrees of freedom of the data and may, unfortunately, lead to downweighting lots of data. However, using lenient thresholds to determine motion-related outliers could lead to increased false positives due to residual motion artifacts uncorrected for in the data. Similarly, robust AR partial correlation though was effective at reducing false discoveries significantly, still had more than expected false positives in the data. Thus, better strategies or multi-step strategies may be needed to reduce the influence of head motion on connectivity.

### 4.4 Impact of Imaging Duration on Resting-State fNIRS

The acquisition rate (sampling frequency, $F_{s}$) and the duration of the resting-state fNIRS scan determine the number of timepoints available for the calculation of connectivity metrics and determine the power and sensitivity of these methods. Longer duration scans may lead to stable measures of connectivity, but in infant and other clinical populations, they may present challenges. However, since the temporal autocorrelation also increases with sampling frequency, higher order models may be necessary in data with higher sampling rate to correct for autocorrelation, else it may lead to increased false positives.^47^ The methods discussed in this study may need to be modified slightly using a higher-order AR models for pre-whitening, if the sampling frequency of the data is higher. Nevertheless, we still expect the patterns of results to hold true, with methods that include pre-whitening outperforming the methods that do not include pre-whitening.

Second, different connectivity metrics may require different imaging durations to achieve stability and probably in different populations. The imaging duration may also depend on the effectiveness of the data preprocessing pipeline. Studies show that scan durations as short as 1 min may be enough to obtain stable and reproducible measures of functional connectivity, and nodal network metrics such as nodal efficiency and nodal betweenness for data acquired at a sampling frequency of 25 Hz in adult populations.^88^ However, longer scan durations (>5  min) may be needed for obtaining stable measures of connectivity measures such as network clustering coefficient, local efficiency and global efficiency.^88^ Similarly, in a sample of children aged 7 to 8 years with fNIRS data were acquired at 50 Hz, an imaging duration of 2.5 to 7 min was needed to obtain accurate and stable functional connectivity and graph-theoretic network metrics.^89^ Furthermore, lower acquisition rate (∼4  Hz as in this study) may require longer duration scans to achieve stability of connectivity measures. A thorough analysis on the impact of the acquisition rate and scanning duration on the stability of fNIRS connectivity metrics is needed. Furthermore, the stability of lagged connectivity measures including Granger causality as well as the extensions and variations discussed in this paper with RS-fNIRS data has not been studied and may warrant further research.

### 4.5 Limitations of the Current Study

This work was primarily a numeric simulation study and, while we introduced structured temporal and spatial noise and motion artifacts as realistically as we could, our findings are nonetheless limited to the properties of our simulations. First, it must be noted that the time series was assumed to be stationary to obtain reliable and valid estimates of connectivity. In real datasets, the use of more complex AR integrated moving average models may be necessary for pre-whitening. Additionally, the stationarity of the time series can be tested by Augmented Dickey–Fuller and Kwiatkowski–Phillips–Schmidt–Shin tests.^19^ The use of scale-less measures such as WTC could lead to more reliable estimates of connectivity if indeed fNIRS time series are non-stationary. Also, the non-stationarities of the time series could provide interesting insights into the dynamics of functional connectivity across time. Second, in our simulations, we used a Gaussian spatial smoothing kernel to produce spatially correlated noise originating in the skin layer. Spatial heterogeneity in global physiology could reduce the effectiveness of the proposed methods as do the number and location of short-separation measurements. Furthermore, the way the data were simulated, both HbO and HbR contain the same information, so the utility of including both HbO and HbR short-separation channels in the partial-correlation models has not been explored. Analyses with task-based fNIRS data seem to suggest that adding both HbO and HbR short-separation channels in the model is beneficial;^51^ future studies should explore this with resting-state analysis. Future studies should replicate the current analyses using experimental RS-fNIRS data and compare the results with the gold standard RS-fMRI to confirm our conclusions. Often, in resting-state analysis, we are concerned more about connectivity differences between population groups or between different conditions. So future studies should also evaluate how the issues due to autocorrelation and systemic physiology manifest as group differences, or as differences in conditions in functional connectivity and causality.

## 5 Conclusion

Complex numerical simulation models may theoretically capture more information about the relationship between fNIRS time channels, but in practice solving them is difficult. In the absence of head motion, use of robust methods may be both computationally inefficient and suboptimal as they can reduce the degrees of freedom. Similarly, although methods such as modified Granger causality can model both the zero-lag and the time-lagged relationships, pre-whitened partial correlation performs best when connectivity information is present at the zero-lag while Granger causality performs best when there is a lagged relationship between time series. The modified MVGC performed well in both scenarios, but at the cost of fewer degrees of freedom and possibly lower sensitivity in situations with low signal-to-noise ratios. Similarly, bandpass filtering, while could probably improve the signal-to-noise ratio and sensitivity, it could also lead to lost degrees of freedom and increased autocorrelation, which may offset the gains. Hence, careful consideration of the steps in the preprocessing pipeline is suggested to maximize sensitivity while still reducing false positive rates to near expected levels.

## Code, Data, and Materials Availability

The MATLAB scripts and functions used to run the simulations supporting the findings reported in this article can be found at https://github.com/pradlanka/rsfc-fnirs. The scripts utilizes the functionality of the NIRS Brain AnalyzIR toolbox, which itself can be found at https://github.com/huppertt/nirs-toolbox.
